# Effects of sublethal methylmercury and food stress on songbird energetic performance: metabolic rates, molt and feather quality

**DOI:** 10.1242/jeb.246239

**Published:** 2024-07-05

**Authors:** Claire L. J. Bottini, Rebecca E. Whiley, Brian A. Branfireun, Scott A. MacDougall-Shackleton

**Affiliations:** ^1^ The University of Western Ontario, Department of Biology, 1151 Richmond St., London, ON, Canada, N6A 5B7; ^2^ Advanced Facility for Avian Research, University of Western Ontario, London, ON, N6G 4W4, Canada; ^3^ The University of Western Ontario, Department of Psychology, 1151 Richmond St., London, ON, N6A 5C2, Canada

**Keywords:** Seasonal environmental changes, Physiology, Energetic cost, Stressors, MeHg contaminant, Avian

## Abstract

Organisms regularly adjust their physiology and energy balance in response to predictable seasonal environmental changes. Stressors and contaminants have the potential to disrupt these critical seasonal transitions. No studies have investigated how simultaneous exposure to the ubiquitous toxin methylmercury (MeHg) and food stress affects birds' physiological performance across seasons. We quantified several aspects of energetic performance in song sparrows, *Melospiza melodia*, exposed or not to unpredictable food stress and MeHg in a 2×2 experimental design, over 3 months during the breeding season, followed by 3 months post-exposure. Birds exposed to food stress had reduced basal metabolic rate and non-significant higher factorial metabolic scope during the exposure period, and had a greater increase in lean mass throughout most of the experimental period. Birds exposed to MeHg had increased molt duration, and increased mass:length ratio of some of their primary feathers. Birds exposed to the combined food stress and MeHg treatment often had responses similar to the stress-only or MeHg-only exposure groups, suggesting these treatments affected physiological performance through different mechanisms and resulted in compensatory or independent effects. Because the MeHg and stress variables were selected in candidate models with a ΔAICc lower than 2 but the 95% confidence interval of these variables overlapped zero, we found weak support for MeHg effects on all measures except basal metabolic rate, and for food stress effects on maximum metabolic rate, factorial metabolic scope and feather mass:length ratio. This suggests that MeHg and food stress effects on these measures are statistically identified but not simple and/or were too weak to be detected via linear regression. Overall, combined exposure to ecologically relevant MeHg and unpredictable food stress during the breeding season does not appear to induce extra energetic costs for songbirds in the post-exposure period. However, MeHg effects on molt duration could carry over across multiple annual cycle stages.

## Teaser

**Summary:** Songbirds under combined exposure to methylmercury and unpredictable food stress during the breeding season do not appear to experience extra energetic costs, but methylmercury effects on increasing molt duration are concerning.

## INTRODUCTION

Organisms regularly modify their physiology and energy balance to maintain homeostasis and adjust to predictable seasonal environmental variation. Because energy is limited, an organism's physiological performance and trade-off in energy allocation strongly influence its survival across life history stages or its lifetime reproductive success (Burton et al., 2011; Vallverdú-Coll et al., 2015; Williams, 2018). Hence, the physiological performance of wildlife is tightly regulated to not waste energy that may be needed later. Unpredicted energetic costs can have carry-over effects from one life history stage, or annual cycle phase, to another and thus impact animal performance long after the initial cause of the cost has disappeared (reviewed in Harrison et al., 2011; Moore and Martin, 2019; O'Connor et al., 2014). For example, a reduced feather length or damaged feather resulting from costs experienced during molt could hinder flight ability and increase flight cost, and thus negatively affect a bird's energy stores throughout the whole year until the next molt (Echeverry-Galvis and Hau, 2013; Jones and Ward, 2020; Swaddle et al., 1996). Energy management and balance are likely under strong natural selection and are associated with fitness-related traits such as movement and activity, boldness and dominance (Arnold et al., 2021; Mathot et al., 2019).

Metabolic rates play a central role in regulating energetic performance and resource allocation. Metabolic rates vary seasonally (reviewed in McKechnie et al., 2015; Swanson, 2010) in response to energy demands and to numerous other biotic and abiotic factors (reviewed in Konarzewski and Ksiazek, 2013; White and Kearney, 2013). For example, metabolic rates change with chick rearing load (Welcker et al., 2015). Ultimately, metabolic rates influence survival and reproductive outcome in birds (Jimeno et al., 2020; Rønning et al., 2016). Basal metabolic rate (BMR) is the adult minimal energy expenditure during rest in a post-absorptive state at thermoneutral temperature (McNab, 1997) and represents an animal's basic maintenance costs (Swanson et al., 2017). Maximum metabolic rate (MMR) corresponds to the maximum aerobic energy expenditure reached during locomotive exercise. As BMR and MMR result from the activity of different tissues (i.e. internal organs and skeletal muscles, respectively; Chappell et al., 1999; Daan et al., 1990; Petit et al., 2014), they represent different ecologically relevant traits of physiological performance (reviewed in Husak and Lailvaux, 2017; Pettersen et al., 2018). The range between MMR and BMR is an individual's metabolic scope, corresponding to the organism's energy pool available for aerobic activity above maintenance level. In other words, it is the organism's capacity to increase its energy consumption for sustained physical performance (Husak and Lailvaux, 2017).

Molt is an important seasonal phase of the annual cycle in birds. Molt efficiency may impact plumage quality such as feather mass (Dawson, 2004; Marzal et al., 2013; Murphy et al., 1988), and losing feathers reduces flight performance (Echeverry-Galvis and Hau, 2013; Guillemette et al., 2007; Swaddle and Witter, 1997a) or survival (Jones and Ward, 2020; Møller and Nielsen, 2018; Slagsvold and Dale, 1996). Once or twice a year, depending on the species (reviewed in Kiat et al., 2019), the flight feathers are dropped and sequentially replaced, inducing an energetic cost (Fox et al., 2014; Murphy and Taruscio, 1995). As a result of tissue growth, poorer insulation and subsequent increased thermoregulatory costs, the metabolic rate of birds increases during molt compared with that in the non-molting period (Lindström et al., 1993; Lustick, 1970; Portugal et al., 2007). Molt may create a 9.8–80% increase in BMR depending on the species (Buttemer et al., 2019; Murphy and King, 1992; Vézina et al., 2009). As a consequence of this cost, molting geese may lose 22–25% of their pre-molt body mass (Fox and Kahlert, 2005; Portugal et al., 2007). However, energy stored before molt and other factors may influence molt performance. For example, food availability affects the duration of molt in birds (Cristol et al., 2014; Danner et al., 2015; Pap et al., 2008) and body condition is positively related to the length of the ninth primary flight feather in geese (Marmillot et al., 2016). Additionally, songbirds that overlapped reproduction and molt produced shorter and lighter feathers (Echeverry-Galvis and Hau, 2013). Overall, energetic trade-offs and carry-over effects can influence birds' molt performance and feather quality.

Exposure to contaminants may disrupt energy acquisition and allocation, through depuration costs, homeostasis dysregulation or behavioral modification (Goodchild et al., 2019). This disruption may reduce energy available for seasonal transitions and physiological performance. For example, methylmercury (MeHg) is a ubiquitous neurotoxin (Henny et al., 2002; Hoffman et al., 2009; Scoville et al., 2020) created from inorganic mercury by bacterial activity in anaerobic habitats (Boyd et al., 2017; Merritt and Amirbahman, 2009; Weber, 1993). Once produced, MeHg bioaccumulates in organisms and biomagnifies along the food chain (reviewed in Mahbub et al., 2017; Munthe et al., 2007), resulting in concentrations of concern at upper trophic levels such as birds (reviewed in Ackerman et al., 2016; Chételat et al., 2020). In birds, MeHg exposure increases BMR and decreases MMR, thus decreasing the metabolic scope of songbirds (Gerson et al., 2019). MeHg also increases birds' molt rate (Carlson et al., 2014), and affects feather quality such as color or mass (Evers et al., 2008; Giraudeau et al., 2015; White and Cristol, 2014). Exposure to MeHg is known to produce carry-over effects on bird survival (Heddle et al., 2020; Ma et al., 2018), reproductive success (Paris et al., 2018), migratory behavior and hormones (Bottini et al., 2022). But the effects of MeHg on birds' energetic performance and seasonal transitions are mainly undetermined. Because MeHg presence in the environment is predicted to increase (Jonsson et al., 2017; Krabbenhoft and Sunderland, 2013; Schaefer et al., 2020), it becomes increasingly important to better evaluate its effects on humans and wildlife.

Like MeHg, stressors may increase individuals' energetic needs and affect molt and feather quality. For example, low food availability can reduce feather growth rate (Bateson et al., 2021; Murphy et al., 1988; Swaddle and Witter, 1997b) or delay molt start (Dawson, 2018), while low-quality diet can increase molt duration (Pap et al., 2008) or may stop molt altogether (Murphy et al., 1988; Scheiman and Dunning, 2004). In contrast, food-supplemented birds have an earlier molt onset or peak (Cristol et al., 2014; Danner et al., 2015; Vaucoulon et al., 1985). Food reduction typically decreases BMR (Brzȩk and Konarzewski, 2001; Rønning et al., 2009; Zhang et al., 2018), but sometimes increases it (Schmidt et al., 2012) and decreases MMR (Moe et al., 2005). Overall, chronic stress may lead to sustained glucocorticoid secretion, resulting in changes in energy allocation (Fokidis et al., 2012; Schoenle et al., 2018), reducing feather regrowth rate (Romero et al., 2005) and feather quality (DesRochers et al., 2009; Murphy et al., 1988) and leading to carry-over effects (Koren et al., 2012; Latta et al., 2016).

In this study, using a 2×2 experimental design, we assessed how exposure to unpredictable food stress and/or an environmentally relevant dose of MeHg affects avian physiological performance. We hypothesized that the effects of stress and MeHg exposure could accumulate and affect birds' energetic performance. We predicted that combined exposure to stress and MeHg would have a greater effect on energetic performance (i.e. increasing BMR while reducing MMR, metabolic scope and feather quality) and seasonal transitions (i.e. decreasing body condition and molt duration) than each of these challenges alone. Detailed results on blood total mercury (THg) accumulation and depuration throughout the experiment have been published in Bottini et al. (2021). Briefly, blood THg reached a maximum value of 5.84±1.48 mg kg^−1^ w/w (mean±s.d.) on week 10 and then plateaued up to the end of exposure on week 12 before molt induced a rapid clearance of most THg within the first 4 weeks post-exposure. In comparison, unexposed birds kept a low blood THg level of 0.0045±0.0058 mg kg^−1^ throughout the experiment.

## MATERIALS AND METHODS

### Bird capture and housing

We captured 49 song sparrows, *Melospiza melodia* (Wilson 1810) (10 females, 39 males), in and near London, ON, Canada (42°59′05.6″N 81°14′43.1″W) using mist nets in two capture sessions. Thirty-one (8 females, 23 males) were captured between 8 August and 1 September 2017 and held overwinter for unrelated experiments (Grieves et al., 2019a; Grieves et al., 2019b) before being transferred to this study, while 18 (2 female, 16 males) were captured between 9 and 11 April 2018. Birds in this study also had preen oil collected for a parallel study (Grieves et al., 2020). All applicable international, national and/or institutional guidelines for the care and use of animals were followed. Birds were captured under permission from Environment and Climate Change Canada permit CA-0244, and all housing and experimental procedures were approved by the University of Western Ontario Animal Care Committee (protocol 2017-161).

We housed birds in individual cages indoors with a relative humidity of 30–70% and temperature of 20–22°C at the Advanced Facility for Avian Research (AFAR), University of Western Ontario. We kept the birds under a photoperiod updated weekly to match those of London, ON, Canada, to maintain their circannual rhythm. Birds had access to a bath cup twice a week and *ad libitum* water and food (Living World Premium Mix for Budgies parakeet seed mixed with ground Mazuri small bird diet). Starting on 16 April 2018, we began to feed them a handmade, nutritionally complete synthetic agar-based diet, which became the birds' main food on 30 April 2018. The dry mass of this diet contained 60% carbohydrate, 13.4% protein and 10.6% lipid (see details in Grieves et al., 2020). Additionally, once a week, we gave the birds about 6 g of blended commercial chicken eggs or 2–4 mealworms to serve as treats.

**Fig. 1.**
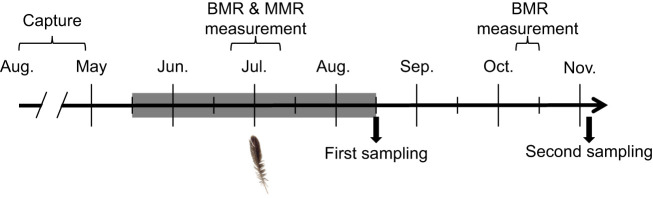
**Experimental timeline.** Gray highlighted area indicates the period of food stress and/or methylmercury (MeHg) exposure. The feather indicates the start of the molt examination carried out weekly until the end of the experiment. Body mass was measured every 2 weeks (not depicted). During the first sampling, 2–4 birds per treatment were taken for another study. BMR, basal metabolic rate; MMR, maximum metabolic rate.

We assigned each bird to one of four treatment groups, balancing for sex and capture session: control (*n*=12: 2 females, 10 males), unpredictable food stress only (*n*=12: 2 females, 10 males), MeHg only (*n*=12: 3 females, 9 males), and combined exposure to food stress and MeHg (*n*=13: 3 females, 10 males). We staggered the start of food stress and MeHg exposure by 24 h such that half of the birds in each of the four groups started treatment on day 1 and the other half started treatment on day 2. We began food stress and MeHg exposure on 15–16 May 2018, and the treatment lasted 90 days up to 13–14 August 2018. Two birds unexpectedly died during this period. Following the treatment period, 17 birds were haphazardly selected from the treatment groups balancing for sex and capture session, and were euthanized via isoflurane inhalation for a different study. We fed the remaining 32 birds (8 in each treatment) the uncontaminated agar diet and water *ad libitum* during a post-exposure period until they were euthanized between 31 October and 4 November 2018 (Fig. 1).

Birds undergoing unpredictable food stress treatment (food stress or combined exposure to MeHg and food stress) had all food removed from their cages for 3 h daily at randomly selected times during the daylight period. During the exposure period, we dosed the agar-based diet with methylmercury chloride (5 mg kg^−1^ wet mass; Alfa Aesar, #33553) to a concentration (mean±s.d.) of 0.19±0.022 mg kg^−1^ wet mass total mercury (THg; concentration corrected for dry mass: 0.58 mg kg^−1^ THg). This dose is within the levels quantified in songbird prey items of mercury-contaminated areas in North America (Cristol et al., 2008; Harding et al., 2006; Newman et al., 2011).

### Blood sampling and THg analysis

We took an initial blood sample from all birds on 1–2 May 2018, before the experiment started, in order to confirm the birds' low initial mercury levels. Then, starting on 16–17 May 2018, we took a blood sample once every 4 weeks. To collect blood samples, we punctured the wing vein with a needle and collected 50–200 µl of blood into heparinized microhematocrit tubes. If a sample could not be centrifuged quickly, it was kept on ice or refrigerated. Within 5–90 min of collection, we transferred 25–50 µl of whole blood into microcentrifuge tubes while the rest was centrifuged to separate plasma from red blood cell. Each sample was then stored at −80°C until analysis.

To measure the THg content of food, we sampled 4 g of each batch of food made during the exposure period and froze it at −80°C until analysis. Blood mercury burden is almost entirely in the form of MeHg (Rimmer et al., 2005; Thompson and Furness, 1989); therefore, we measured THg content and used this value as an index of bird MeHg burden. We performed THg analysis at the Biotron (an ISO 17025 accredited facility) at the University of Western Ontario, Canada. We used a Direct Mercury Analyzer (DMA-80, Milestone Inc., Shelton, CT, USA) following US EPA Method 7473 (EPA, 1998). Methods for mercury analysis of food and blood samples are provided in Bottini et al. (2021).

### Body condition, fat and lean mass

Before the experiment, we measured each bird's tarsus length to the nearest 0.1 mm using dial calipers. We also measured each bird's body mass with an electronic balance to the nearest 0.01 g every 2 weeks from the beginning to the end of the experiment. We calculated body condition as body mass (g) divided by tarsus length (mm) (see Table S1 legend). This resulted in a continuous variable with higher values corresponding to birds with higher mass relative to their size. While most of the variation in body condition is driven by body mass, the use of a ratio permitted a better comparison of seasonal changes in morphology across birds with different tarsus sizes (mean±s.d. tarsus length 21.75±0.71 mm, range 20.1–23.5 mm; *n*=49). Indeed, mass and mass changes through time were both positively correlated with tarsus length (see Table S1).

On the same day that body mass was measured, we also quantified fat and lean mass using quantitative magnetic resonance (EchoMRI-B, Echo Medical Systems, Houston, TX, USA; Guglielmo et al., 2011). Measurements were taken in duplicate using ‘small bird’ option and ‘two accumulations’ setting with water stage off and were then averaged. To improve the accuracy of measurements, we used a calibration equation (fat mass: raw value×0.94; lean mass: raw value×1.02) following prior studies (Guglielmo et al., 2011; Schmidt et al., 2012).

### Metabolic rates

We measured metabolic rates using open-circuit respirometry, following previously established methodology (Schmidt et al., 2012). We first measured the bird's ‘minimal’ energy expenditure (i.e. BMR). Second, we evaluated their MMR during short-term (20–30 min) exercise. The same air flow system was used to determine the BMR and MMR of each bird.

We measured the BMR of birds between 21 June and 5 July 2018 (weeks 6–8, during the treatment exposure period) and from 7 to 16 October 2018 (weeks 22–23, during the post-exposure period). Each night, 4-5 birds were placed in a stainless-steel chamber in a temperature-controlled cabinet at 30°C, while a sixth chamber was used for baseline measurements. A temperature of 30°C is within the thermoneutral zone for other species of songbirds that are similar in size to song sparrows (Root et al., 1991). In June, the birds were fasted in their home cage from 19:00 h, and we measured body mass beginning at 19:30 h before placing the birds into a chamber. We started to record the birds' O_2_ consumption and CO_2_ emission around 20:15 h and stopped approximately 10 h later, around 06:45 h. In October, because the light schedule changed, the birds were fasted in the chamber instead of their home cages. We took body measurements beginning at 18:30 h, and recording started around 19:00 h to finish around 07:15 h the next morning (approximately 12.25 h later). Following this, we measured each bird's body mass again and returned the birds to their home cage, where they were left undisturbed for one full day (no food stress treatment).

To measure BMR, the incurrent air was scrubbed of CO_2_ and water vapor using soda lime and Drierite (W. A. Hammond Drierite Company, Xenia, OH, USA), respectively. The six sealed chambers received a constant air flow of 450 ml min^–1^. Excurrent air was sub-sampled at 150 ml min^–1^. This air passed through a Drierite column to remove water before going into the CO_2_ analyzer (catalog number CA-2A, Sable Systems International, Las Vegas, NV, USA) and the O_2_ analyzer (FC-1B, Sable Systems International). Gas analyzers were calibrated regularly using a standard containing 20.9% O_2_ and 2% CO_2_ balanced with N_2_. Using a multiplexer (Sable Systems International), chambers were measured one after the other, multiple times throughout the night. The baseline chamber was measured for 5 min, and a bird's chamber was measured for 10 min before switching to the next chamber. All instruments were connected to an analog-to-digital converter (UI-2 model, Sable Systems International), which was connected to a laptop computer. During the night of recording, each bird had 11 measurements of 10 min recorded in June–July and 13 measurements recorded in October.

Data were analyzed using Expedata version 1.7.2 (Sable Systems International). Out of the multiple 10 min recordings, we extracted the minimum 5 min mean of O_2_ consumption and reported this value as BMR. We calculated the rate of O_2_ uptake (*V̇*_O_2__) based on equation 10.6 in Lighton (2008), which calculates *V̇*_O_2__ using the data for both O_2_ consumption and CO_2_ production. We then converted *V̇*_O_2__ to watts using eqn 9.13 and associated text in Lighton (2008):
(1)

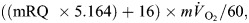
where mRQ is the mean respiratory quotient (defined as the ratio of CO_2_ emitted to O_2_ consumed) during the 5 min period. In order to compare the bird in a post-absorptive state, we discarded the metabolic rate data collected within the first 3 h of the beginning of fasting. As a reminder, the birds fast started at 19:00 h in June and when entering the chamber around 18:45 h in October, hence they finished their fasting within the chamber.

We measured MMR 2 days before or up to 6 days after each bird's BMR measurement, between 28 June and 8 July 2018 (during the treatment exposure period). We measured MMR using an enclosed exercise wheel (16×24 cm, width×diameter) made of acrylic plastic and lined with rubber that induced birds to actively hop and hover while the wheel rotated, following prior research (Pierce et al., 2005; Price and Guglielmo, 2009; Schmidt et al., 2012). Air flowed into the wheel at a rate of 4000 ml min^–1^ and was subsampled as described above for measurements of BMR. Three ping-pong balls were placed in the wheel to prevent birds from walking.

Food dishes were removed 3 h before testing (from 07:30 h to 10:30 h) to ensure that birds were in a post-absorptive state. Beginning at 10:30 h and finishing no later than 14:00 h, we measured MMR in 3–6 birds each day. Before and after each metabolic measurement, we noted the bird's body mass to quantify water loss but we later used the mass when exiting the chamber in subsequent analysis. Once the bird was placed into the wheel, we covered it with a blanket and allowed the bird to acclimate for 10 min. We then removed the cover and spun the wheel manually to initiate exercise. The wheel was kept in constant motion so that the birds were forced to hop and hover until MMR was reached (this always occurred within 30 min). During the exercise, we noted how active the bird was. In all cases, after MMR was reached, O_2_ consumption decreased and then stabilized. The MMR of an individual was calculated as the maximum mean of O_2_ consumption over a 1 min period expressed in watts. We then calculated the factorial metabolic scope as the ratio of MMR to BMR for each individual measured in June to estimate the aerobic capacity range.

### Molt

We monitored molt of the right wing primary flight feathers once every week from early July (during the treatment exposure period) until the completion of the experiment at the end of October. For each primary feather, we recorded whether the feather was old, had fallen or was growing and estimated the growth percentage (on a 0 to 100 scale; method modified from Nolan et al., 1992). We combined the individual scores of the nine primary feathers to create a molt score between 0 and 900.

We determined the start date of each bird's molt as the first week we observed a missing or newly growing primary feather, P1. We deemed the entire molt period had finished when the molt score reached 900. For four birds, some growing feathers were lost as a result of handling, so the molt score was estimated via the percentage growth of other proximate growing feathers. For three birds, the last growing feathers were close to completion (≥70% growth) but not fully grown at the time of euthanasia in November. In these cases, the date of molt completion was estimated to be 1 week after the last measurement. Additionally, two further birds had not started to grow their last two primary feathers before euthanasia, so their date of molt completion was estimated to be 2 weeks after the last measurement. These estimates were based on the observation that most feathers grew within 2–4 weeks. Finally, we calculated the overall molt duration as the number of weeks between molt start and finish.

### Feather quality

**Fig. 2.**
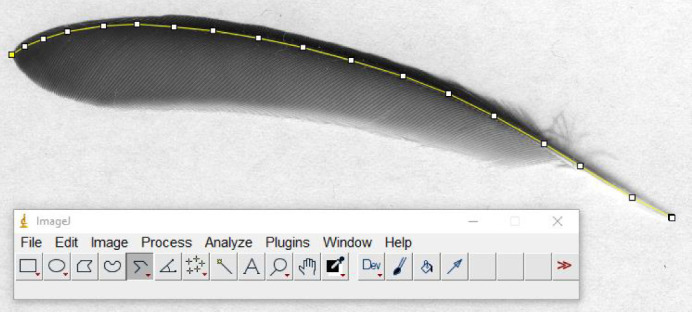
**Scan and measurement of a primary feather (P5) with ImageJ software.** Scans were made at either 800 or 1200 dpi depending on feather number. The yellow line indicates the segmented line tool used to quantify feather length.

We collected newly grown primary feathers post-mortem from the right and left wings of the same 32 birds that were monitored for molt. Primary feathers P1, P2, P5, P7, P8 and P9 from the right wing and P1, P5 and P9 from the left wing of each bird were collected. Each feather was kept in a separate paper envelope and stored at room temperature. While each bird started their molt at different times, for most birds, P1 and sometimes P2 grew during the exposure period while most P5 and following feathers grew during the post-exposure period (more details are provided in Bottini et al., 2021). We measured the mass of each feather with an electronic scale (Sartorius CP224S) to the nearest 0.1 mg before scanning them on a flatbed document scanner (image definition: 800 dpi for right wing P2, P5, P7; 1200 dpi for right wing P1, P8, P9 and left wing P1, P5, P9). We measured the curved length from the tip of the quill up to the end of the rachis, following the feather rachis (Fig. 2), to the nearest 0.1 mm, with ImageJ software and segmented line tool (scale set at either 31.4 or 47.2 pixels mm^–1^). Mass data from feathers still partly in sheath were discarded (*N*=25/288). Feather length data were discarded if the molt data indicated that the feather had not finished growing (*N*=18/288). From the remaining data, we then calculated the mass:length ratio to use in further analyses. We also quantified the difference in length between right and left primary feathers P1, P5 and P9 as an estimate of wing asymmetry.

### Statistical analysis

All statistical analyses were done using R version 4.0.3 (http://www.R-project.org/), and a significant threshold of α=0.05. Data are reported as means±s.d. For all the models tested in this study, we used similar statistical protocols, aiming to identify influential variables for our data while not oversaturating the models we were working with. First, we performed preliminary analysis to determine the random structure (e.g. one random intercept, correlated random intercept and slope, uncorrelated random intercept and slope, or two random intercept structures) and whether a time variable needed to be fitted with a polynomial function because of its non-linear effect on our measurement. We did this through model comparison using Akaike's information criterion corrected for small sample sizes (AICc) and the *anova* function on initial models including only the variables of interest from our *a priori* hypothesis (i.e. 2- or 3-way interaction of MeHg×food stress with time when needed). In a second step of the preliminary analysis, using the same model comparisons method, we assessed whether variables associated with low sample size (i.e. sex; females *n*=10 during the exposure period and *n*=5 during the post-exposure phase) or variables with no *a priori* expectation (e.g. tarsus length, date of the metabolic measurements, capture session, water loss) were influencing the data and needed to be included or not in the model. With this information, we created a ‘saturated model’ incorporating our variables of interest and the other influential variables highlighted during preliminary analysis. We then used the *dredge* function in *MuMIn* (https://CRAN.R-project.org/package=MuMIn) and AICc to compare the support for alternative simpler models through an information-theoretic approach (Anderson and Burnham, 2002). Candidate models differed in the presence and absence of terms compared with the saturated model. If several candidate models had a ΔAICc<2, we compiled their model-averaged trait estimates using the conditional averaging method (Anderson and Burnham, 2002) implemented with the *model.avg* function of the *MuMIn* package. For linear mixed effect models (lme), we used the *lmerTest* package (https://cran.r-project.org/web/packages/lmerTest/index.html) (Kuznetsova et al., 2017), and the results were obtained by fitting the model with restricted maximum likelihood, and each model *R*^2^ was extracted via the *r.squaredGLMM* function of the *MuMIn* package. In all models including multiple sampling through time, we chose to analyze the data with lme and a polynomial function instead of generalized additive mixed models (*gamm*) as this function currently does not permit tests for 3-way interactions between factorial and continuous variables, preventing us from assessing our *a priori* hypothesis. The dataset and R code for the full data analysis are available from Mendeley data repository (doi:10.17632/knbpvd4yyk.2).

#### Fat and lean mass

To analyze changes in body condition, fat and lean mass and over time, we used the first measure taken prior the start of treatment exposure as a reference point to quantify the mass gained or lost with time (the number of weeks since the start of treatment exposure). This measure of change accounts for the initial difference in body condition between sex and between some of the treatment groups (see Table S1 legend). Statistical analysis of the change in body condition is presented in Table S1 and Fig. S1.

For the fat mass analysis, our saturated lme model included the triple interaction of food stress, mercury and a quartic polynomial function for time as fixed effects. Time and bird ID were included in the model as correlated random intercept and slope to account for the repeated measures.

A similar model was used for lean mass data analysis, except that time was better explained by a cubic polynomial function and tarsus was not included in the model. Time and bird ID were also included in the model as two random intercepts. We used the same procedure to remove irrelevant fixed effects and extract results from the final model.

#### Metabolic rates

In order to reduce the number of covariates in the models, we chose to study metabolic performance (for BMR and MMR) using mass-corrected data. We used body mass instead of other measures of structural size (e.g. tarsus length, body condition) as it resulted in a better linear fit for both BMR and MMR (see results in R code: doi:10.17632/knbpvd4yyk.2) and accounted for the change in muscle mass over time. To do this, we first used regression analyses relating log_10_ transformed metabolic rate (in W) with log_10_ transformed body mass measured when birds exited the metabolic chamber. For BMR data, this linear regression was done for each measurement period separately (i.e. in June–July during exposure and in October during post-exposure). The log_10_ transformation was done as the relationship between mass and metabolic rates of animals is typically non-linear. We used this regression to calculate the scaling equation for both BMR and MMR. Residuals of the regression were then extracted and used in further analysis.

For BMR analysis, lme model residuals violated homogeneity assumptions, and no data transformation or addition of fixed factors improved the model fit. Therefore, we instead used a non-parametric Kruskal–Wallis test on each measurement period separately (i.e. exposure versus post-exposure) to analyze the mass-corrected BMR residual variation between treatments. If a significant effect of treatment was found, we followed up the analysis with a Kruskal–Wallis multiple comparison test as a *post hoc* assessment (Dunn, 1964) as it is appropriate for groups with unequal observations, and used the default Holm method of *P*-value adjustment. Because we were also expecting changes in measurements with seasonal transition, we also checked whether the measurement period affected the mass-corrected BMR residuals of birds kept until November via a non-parametric Wilcoxon test. We chose this test instead of a parametric paired *t*-test as the data were not homogeneous between month groups. Finally, several birds were still molting at the time of the October BMR measurement. We thus used a *t*-test to assess whether the state of molt (finished, *n*=13; ongoing, *n*=19) affected the BMR residual values of those birds.

Before starting the analysis of MMR data, we removed four birds (two from the control and two from the combined exposure treatment) that were not exercising in the wheel (e.g. sliding instead of actively hopping and hovering) from the dataset. Then, for consistency with the BMR analysis above, we analyzed the MMR mass-corrected residuals variation, using a linear model (lm) including the interaction between food stress and mercury treatment, and the date of the measurement (number of days since the first day of MMR measurement) as independent variables.

Before starting the analysis of metabolic scope, we removed the same four birds that did not perform well during MMR measurement from the analysis. We then assessed the effects of treatment on factorial metabolic scope (MMR:BMR) via a lm, with the interaction between food stress and mercury, and the difference in number of days between BMR and MMR measurement as an independent effect. One data point was identified as influential using the influence.measures function of the *stats* package (http://www.R-project.org/) with a covariance ratio <1. We therefore chose to present the model without this data point but keeping it would have resulted in the food stress effect becoming significant (*P*=0.027). Similar analysis was done for absolute metabolic scope (MMR−BMR); the model is presented in Fig. S2 (see legend).

#### Molt

First, we checked the correlation between the date of molt start, molt duration and date of molt end via Spearman rank correlation analysis. We chose a non-parametric test as these measurements were not normally distributed. Then, we decided to focus on molt duration only as this integrates information from molt start and end. The analysis for molt start and end is presented in Table S2. Molt duration was analyzed via lm including the interaction between food stress and mercury as well as sex as fixed effects.

#### Feather quality

We first assessed feather quality via their mass:length ratio calculated for primary feathers P1, P2, P5, P7, P8 and P9 of the right wing. The saturated lme model included the triple interaction of MeHg, food stress exposure and primary feather number as fixed effects and bird ID as a random intercept. We also did the same analysis on feather length and mass, as dependent variables (see Table S3).

In the second step, we determined whether the treatments affected the asymmetry of feather length. We calculated the feather P1, P5 and P9 absolute difference in length (mm) between the right and left wings. This difference was square-root transformed and then assessed within a lme model including the triple interaction of MeHg, food stress exposure and primary feather number as fixed effects and bird ID as a random intercept. One bird with only one value was removed from the dataset before analysis.

## RESULTS

### Fat and lean mass

**Fig. 3.**
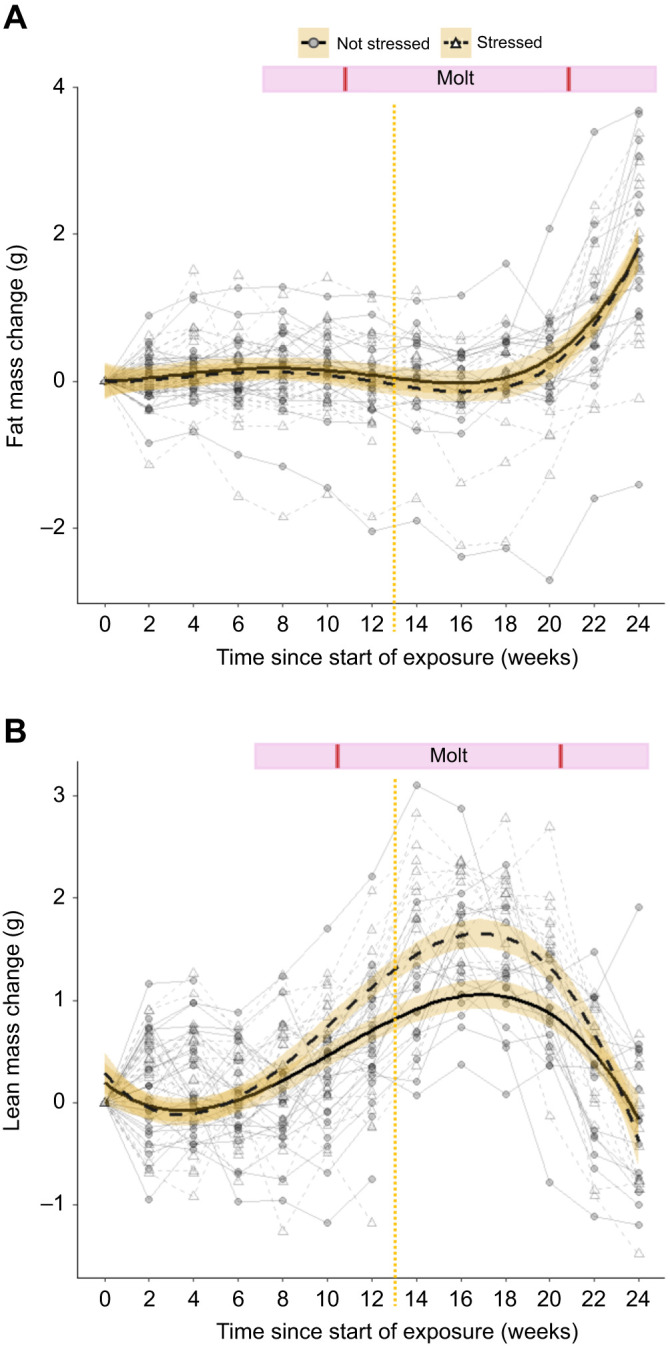
**Change in fat and lean mass over time, according to food stress treatment.** (A) Fat mass and (B) lean mass (means±s.e.m.). Time point 0 corresponds to the pre-experiment body condition measured on 14–15 May. The vertical yellow dotted line indicates the end of treatment exposure (when 2–4 birds per treatment were removed for a separate study). The horizontal blue bar indicates the start and end of the molt period (primary feathers), with vertical red lines indicating the mean date of molt start (3 August) and molt end (12 October). Regression lines were fitted via a lm with a quartic and cubic polynomial function, respectively. The solid line and filled circles (*n*=16 by the end of experiment) indicate birds not exposed to food stress (control and MeHg-only treatments), while the dashed line and open triangles (*n*=16) indicate birds exposed to food stress (food stress-only and combined MeHg and food stress treatments). Connected symbols represent individual bird ID.

We assessed how fat and lean mass changed with time and experimental treatments. Of the candidate lme models predicting fat change during the experiment, the best supported model only included the main effect of time with its quadratic function (Table 1A). All birds had low variation in fat mass during most of the experiment but strongly increased in fat mass during the last three time points of measurements, corresponding to the migratory period in October (Fig. 3A). Two other models were within 2 ΔAICc units of this top model; both included the effect of time and the main effect of either food stress or MeHg exposure. However, the parameter estimates for food stress and MeHg exposure were negative and overlapped zero, indicating that treatment exposures had no simple main effect on the change in fat mass during the experiment.

**Table:**
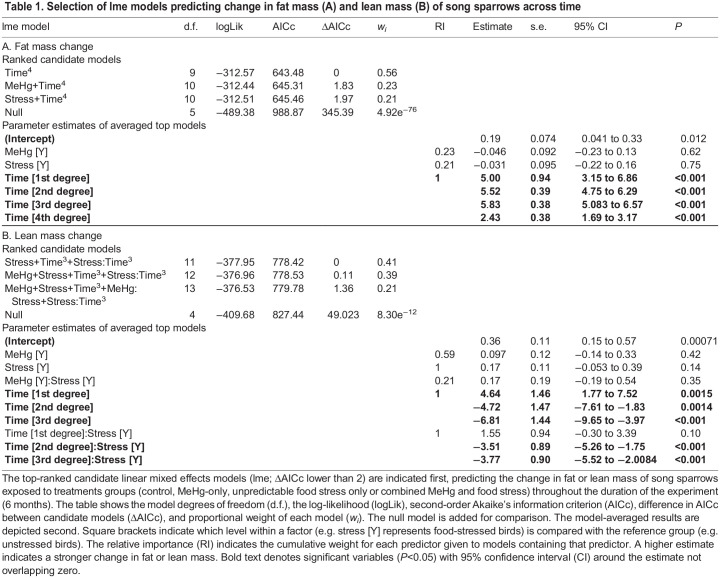
Table 1. Selection of lme models predicting change in fat mass (A) and lean mass (B) of song sparrows across time

The best supported candidate lme model predicting lean mass change during the experiment included the main effect of food stress, the main cubic effect of time and the interaction between time and food stress treatment (Table 1B). All birds slightly decreased in lean mass during the first couple of weeks of the experimental exposure and then increased in mass, with a peak in the weeks of 3 to 17 September (post-exposure period), before decreasing until the end of the experiment (Fig. 3B). Stressed birds showed a greater gain in lean mass during the exposure and post-exposure periods compared with unstressed birds, but became similar to them during the last two measurement time points (Fig. 3B). Two other models were within 2 ΔAICc units of this top model, all including the same variables as the top model in addition to the main effect of MeHg exposure and the MeHg×food stress interaction for the last model. The parameter estimates for MeHg exposure and its interaction with food stress were positive but overlapped zero, indicating weak evidence that these effects affected the change in lean mass during the experiment. Changes in body condition integrated the results from both fat and lean mass change (see Table S1, Fig. S1).

### Metabolic rates

**Fig. 4.**
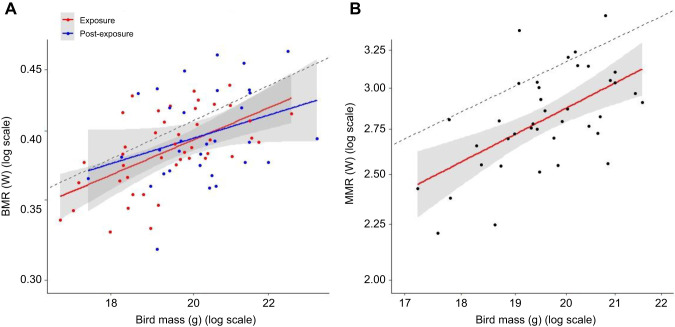
**Variation of metabolic rates according to mass when exiting the chamber.** (A) Basal metabolic rate (BMR) during exposure (June–July, *n*=44) or post-exposure (October, *n*=32). A dashed line with a slope of 0.7 was added for comparison to allometric scaling of the metabolic rate with mass (intercept arbitrarily selected for the line to appear in the figure). (B) Maximum metabolic rate (MMR) measured during exposure (*n*=39). A dashed line with a slope of 1 was added for comparison to isometric scaling of the metabolic rate with mass (intercept arbitrarily selected for the line to appear in the figure). Note the different *y*-axis scale in A and B.

**Fig. 5.**
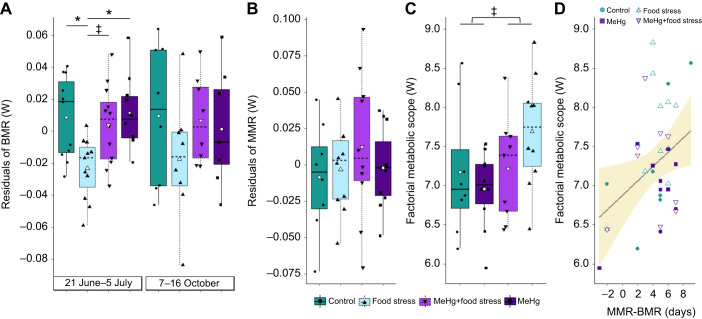
**Variation of metabolic rates according to treatment group and date.** (A) BMR residuals (corrected by mass) during exposure (*n*=10–12 for each treatment) or post-exposure (*n*=8 for each treatment). (B) MMR residuals (corrected by mass) during exposure (*n*=8–11 for each treatment). (C) Factorial metabolic scope (MMR:BMR ratio). Note that the treatment order was changed in this panel to highlight the group differences. Boxplots in A–C indicate the median, 25th and 75th percentiles, and whiskers indicate the range, with individual jittered data points overlaid. Boxplot colors and symbols indicate treatment groups: control, food stress, combined MeHg and food stress, and MeHg. White symbols indicate mean metabolic rate for each treatment group. Significant differences between treatments are indicated (^‡^*P*<0.1 and *P<0.05; Kruskal-Wallis and multiple comparison *post hoc* tests were used for BMR analysis and a lm was used for the other analyses). (D) Effect of the number of days between MMR and BMR measurements on the factorial metabolic scope. Regression line and s.e. shading were fitted via lm.

The BMR to mass scaling equation was *Y*=0.29*M*^0.64^ (log_10_ mass 95% confidence interval, CI=0.36–0.93; adjusted *R*^2^=0.32, *P*<0.001) during exposure in June–July and *Y*=0.37*M*^0.47^ (log_10_ mass 95% CI=−0.033–0.96; adjusted *R*^2^=0.078, *P*=0.066) during post-exposure in October (Fig. 4A). Treatment affected BMR residuals during the time of exposure (Kruskal–Wallis test: χ^2^=10.99, d.f.=3, *P*=0.012; Fig. 5A) but not 2 months post-exposure in October (χ^2^=2.065, d.f.=3, *P*=0.57). During exposure, the BMR residuals from birds in the food stress-only treatment (mean±s.d.: −0.023±0.019 W, *n*=11) were lower than those of birds in the control treatment (0.0086±0.026 W, *n*=11, Kruskal–Wallis multiple comparisons: *P*=0.034) and MeHg-only treatment (0.012±0.023 W, *n*=10, *P*=0.016), but not significantly lower than the co-exposure treatment (0.0038±0.024 W, *n*=12, *P*=0.078). No difference was observed among other treatments groups (*P*>0.1; Fig. 5A). There was no change in BMR residuals between exposure and post-exposure periods in birds kept until the end of the experiment (Wilcoxon signed rank test: *V*=262, *P*=0.98). Furthermore, molting state did not influence BMR residuals of birds in October (*t*-test: *t*=−1.59, d.f.=27.4, *P*=0.12).

The MMR scaling equation was *Y*=0.39*M*^1.08^ (log_10_ mass 95% CI=0.5–1.58; adjusted *R*^2^=0.32, *P*<0.001; Fig. 4B). Of the candidate lm models predicting MMR, the best supported model only included the main effect of measurement date (Table 2A). The last day of MMR measurement appeared to drive this relationship as, if these data points were removed, day was no longer significant (*P*=0.33). Two other models were within 2 ΔAICc units of this top model, each including the main effect of either food stress or MeHg exposure. However, the parameter estimates for food stress and MeHg exposure were positive and overlapped zero, suggesting weak support for treatment effects on MMR (Fig. 5B).

**Table:**
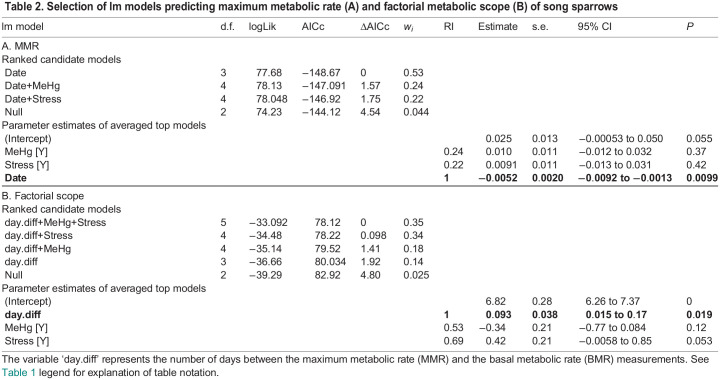
Table 2. Selection of lm models predicting maximum metabolic rate (A) and factorial metabolic scope (B) of song sparrows

The factorial metabolic scope (MMR:BMR ratio) best candidate model included the main effects of MeHg, food stress and the number of days between measurement of MMR and BMR (Table 2B). Three other models within 2 ΔAICc units of this top model also included these variables but in reduced combination. Metabolic scope of birds increased in those with the largest number of days between the two metabolic measures (Fig. 5D), although this trend was driven by one night of measurement, which, if removed or considered as an absolute difference in days, would become non-significant. The parameter estimates for MeHg and food stress were positive but overlapped zero, indicating weak treatment effects on metabolic scope (Fig. 5C). However, this overlap was marginal for food stress (or became significant if the removed influential data point was kept), suggesting a slight increase in factorial metabolic scope in birds exposed to unpredictable food stress (mean±s.d.: 7.47±0.71 W, *n*=19) compared with non-stressed birds (7.05±0.66 W, *n*=18). Furthermore, the absolute metabolic scope (MMR−BMR) was not different between treatments, although the stress effect was retained in two out of the four candidate models with a ΔAICc lower than 2 (see Fig. S2 legend).

### Molt

We first assessed how molt start, duration and end date were related. Spearman correlations indicated that molt start date was positively correlated with molt end date (Spearman: *S*=1773.1; ρ=0.68; *P*<0.001; Fig. S3A), but not with molt duration (Spearman: *S*=6914.9; ρ=−0.27; *P*=0.14; Fig. S3B), while molt duration was positively correlated with molt end date (Spearman: *S*=3085.3; ρ=0.44; *P*=0.013; Fig. S3C). As most measures of molt covaried, we focused on molt duration only.

**Fig. 6.**
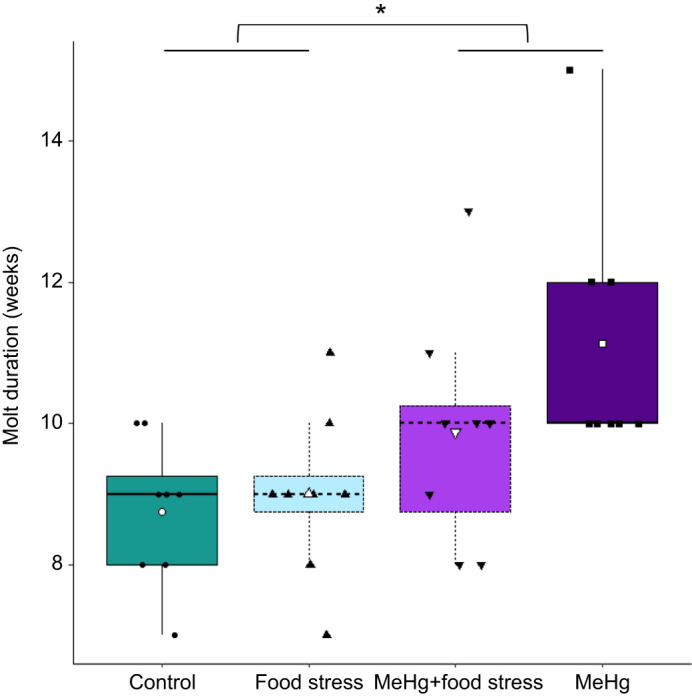
**Effect of food stress and MeHg exposure treatments on molt duration.** Boxplots indicate the median, 25th and 75th percentiles, and whiskers indicate the range, with individual jittered data points overlaid. Boxplot colors and symbols indicate treatment groups: control (*n*=8), food stress (*n*=8), combined MeHg and food stress (*n*=8) and MeHg (*n*=8). White symbols indicate the mean molt duration for each treatment group. The asterisk indicates the lm difference between MeHg treatment groups (*P*<0.05).

Only one candidate model for molt duration was selected via ΔAICc. We thus extracted *F*-values for this model's variables via the *Anova* (type III) function, while the whole model result and *R*^2^ were obtained by the *summary* function. Molt duration was affected by MeHg exposure (lm: *F*_1,29_=9.75, *P*=0.0040; Fig. 6) and sex (lm: *F*_1,29_=5.75, *P*=0.023). MeHg-exposed birds took longer (10.50±1.79 weeks; *n*=16) to molt compared with unexposed birds (8.88±1.09 weeks; *n*=16), while females (11.20±2.39 weeks; *n*=5) took longer to molt than males (9.41±1.39 weeks; *n*=27). The model was significant (lm model: *F*_2,29_=8.46, residuals s.e.=1.38; adjusted *R*^2^=0.33, *P*=0.0013). Despite the significant effect on molt duration, similar analysis on molt start and end demonstrated no treatment effect on these measures, although MeHg was retained in several candidate models with a ΔAICc lower than 2 and had a marginal effect on molt end (see Table S2, Fig. S3D,E).

### Feather quality and wing asymmetry

**Fig. 7.**
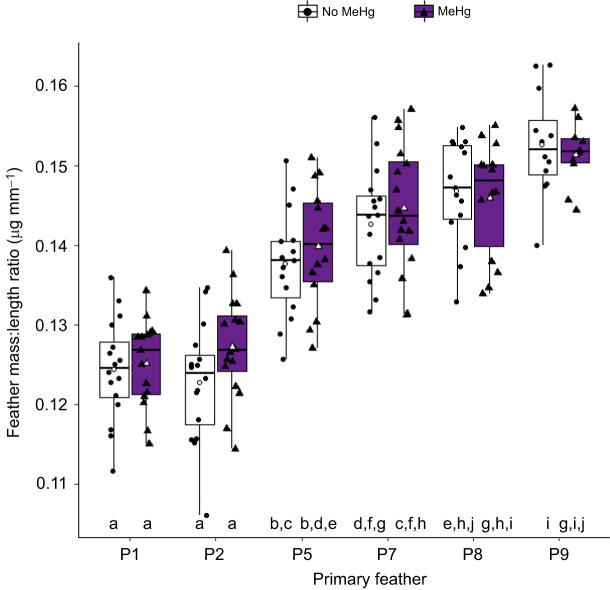
**Effect of MeHg exposure on primary feather mass:length ratio in the right wing.** P1 is the innermost primary feather and, usually, the first primary feather molted (*n*=15–16 per treatment), while P9 is the outer feather (*n*=10–12 per treatment). Boxplots indicate the median, 25th and 75th percentiles, and whiskers indicate the range, with individual jittered data points overlaid. Boxplot colors and symbols indicate the exposure groups: no MeHg (control and food stress only) and MeHg (MeHg only and MeHg+food stress). White symbols indicate the mean body condition for each treatment group. Different lowercase letters indicate significant differences between groups (Tukey *post hoc* test *P*<0.05 from an lme model including the interaction between MeHg and primary feather, with bird ID as random intercept).

We also explored whether exposure to MeHg and food stress affected feather quality. The best candidate models for the feather mass:length ratio included the main effect of primary feather number (Table 3). The feather mass:length ratio increased from P1 to P9. Five other models within 2 ΔAICc units of this top model also included this variable as well as different associations with food stress, MeHg exposure and the interaction of MeHg with primary number. The parameter estimates for MeHg and feather P2 interaction were positive and did not overlap zero, while the overlap included zero for the other feathers. This indicates that inner primaries had greater mass:length ratio in MeHg-exposed birds than in uncontaminated birds but that this difference disappeared in later-grown feathers (Fig. 7). The parameter estimates of the averaged top models for MeHg and food stress were not significant, suggesting weak support for treatment effects on all feathers. Similarly, the interaction between MeHg treatment and primary number was significant for feather mass, but the parameter estimates for the interaction on feather length did not differ from zero, despite being included in the top model (see Table S3).

**Table 3.**
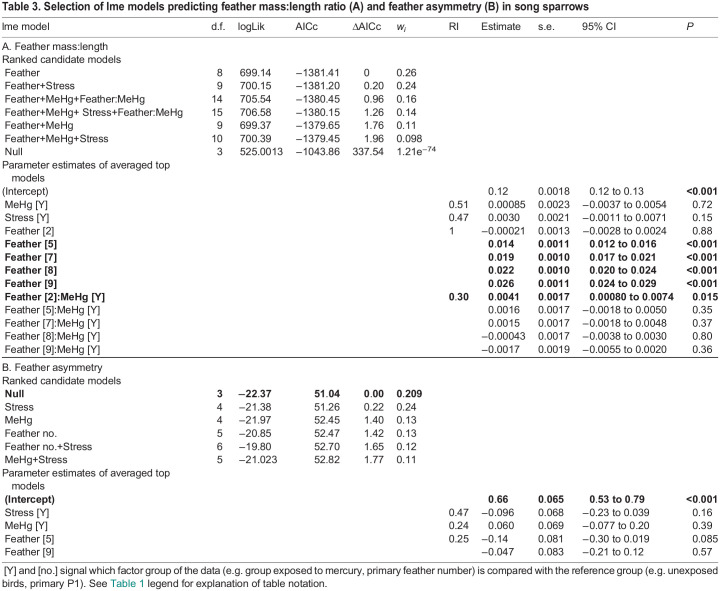
Selection of lme models predicting feather mass:length ratio (A) and feather asymmetry (B) in song sparrows

The best candidate model for the asymmetry in the length of feathers P1, P5 and P9 between the right and left wings was the null model. Thus, there was no detectable influence of treatment or feather type on feather asymmetry (Table 3).

## DISCUSSION

We aimed to assess whether avian physiological performance would be more strongly affected by combined MeHg and unpredictable food stress exposure than by either treatment on its own, and to assess seasonal changes in the birds' physiological response to the treatments. Contrary to our main prediction, combined exposure to MeHg and food stress did not result in additive or synergistic effects. For most measures, birds exposed to both treatments had intermediate responses (BMR) or similar responses (other measures) compared with birds exposed to the food stress-only or MeHg-only groups. It thus appears that each challenge affected the birds via different mechanisms that do not interact. According to prior research, body condition, metabolic rates, molt and feather quality should each be influenced by energetic costs (Daan et al., 1990; Marmillot et al., 2016; Murphy et al., 1988; Portugal et al., 2007; Vézina et al., 2009). However, our study demonstrates an independent effect of unpredictable food stress on body condition and metabolic rate, whereas MeHg exposure primarily affected molt and feather quality. In a parallel study of these same birds, we observed similar compensatory or independent effects on migratory activity and thyroxine levels, but additive effects on fecal corticosterone levels during the post-exposure period (Bottini et al., 2022). Additionally, because the MeHg and food stress variables were selected in candidate models with a ΔAICc lower than 2 but the 95% CI of the variables overlapped zero, we found weak support for MeHg effect on all measures (except BMR), and for food stress effects on MMR, factorial metabolic scope, and feather mass:length ratio. This suggests that MeHg and food stress effects on these measures are statistically identified but too complex and/or too weak to be detected via linear regression. For the rest of the Discussion, we only consider the variables with significant or marginally significant effects as having a biologically relevant impact on the measures. Finally, we encourage the reader to keep in mind our small sample size, so caution is to be taken when interpreting the results and further studies are warranted to confirm our findings.

### From fat and lean mass to body condition

Changes in fat and lean mass with time were differently affected by the treatment exposure and seasons. Surprisingly, fat mass showed little to no change except for the last measurements, when it increased during the period when these birds would be migrating. Lean mass increased during the exposure and post-exposure periods in stressed birds, probably in preparation for migration (Dietz et al., 1999), and then decreased during the last three time points. This decrease could have been caused by the catabolism of muscles during the migratory period (Gerson and Guglielmo, 2011) and resulted in the increase in fat mass in October. Indeed, at this time point, all birds displayed migratory restlessness behavior (Bottini et al., 2022), suggesting that they were in a migratory physiological condition. These effects on fat and lean mass interacted to influence body condition, which was also influenced by stress treatment (Fig. S1). Prior research on the effect of unpredictable food stress on body condition has shown conflicting results. Some studies found no effect (Dall and Witter, 1998; Witter et al., 1995), while others observed an increase (Bednekoff and Krebs, 1995; Cornelius et al., 2017; Cuthill et al., 2000) or a decrease (Acquarone et al., 2002; Fokidis et al., 2012) in fat and body mass. Most research on food stress effects on body condition or mass measured mass during exposure periods only. Also, birds' body mass adjustment strategies can be species and season specific (Witter et al., 1995). However, while lean mass of stressed birds started to differ from that of unstressed birds from about the third month of treatment exposure (week 10 in Fig. 3B), birds exposed to unpredictable food stress had reduced food consumption during the first 2 months of the experiment but not afterward (C. L. J. Bottini, B. Laxer, A. Khaira, B. A. Branfireun and S. A. MacDougall-Shackleton, unpublished). This lack of overlap between food consumption and lean mass suggests that either one is not associated with the other or that the reduced food consumption in stressed birds stimulated a delayed increase in lean mass once they consumed a similar amount of food to unstressed individuals. Hence, more studies on the long-term effects after stress is removed are warranted. Similar to our results, a meta-analysis did not observed modification in body condition in breeding birds under MeHg exposure (Carravieri et al., 2022), while MeHg exposure had no effect on the birds' food consumption (C. L. J. Bottini, B. Laxer, A. Khaira, B. A. Branfireun and S. A. MacDougall-Shackleton, unpublished). Overall, our results indicate that previously stressed birds increased lean mass for migration earlier than unstressed birds, which could lead to an earlier departure for migration, as observed at stop-over sites (Lupi et al., 2016; Schaub et al., 2008; Schmaljohann and Naef-Daenzer, 2011).

### Metabolic rates

We found weak support for MeHg effects on BMR, MMR and metabolic scope. This differs from a prior study where zebra finches (*Taeniopygia guttata*) exposed to 0.6 mg kg^−1^ MeHg for 8 weeks showed an increase in BMR and a reduction in absolute metabolic scope compared with control individuals (Gerson et al., 2019). However, those authors found no significant effect of MeHg on MMR. In our study, the exposure dose was lower than in the zebra finch study, and we measured metabolic rates during the 6th to 7th week of exposure instead of at ≥8 weeks. However, our song sparrows' blood THg levels (mean±s.d. 5.25±0.69 mg kg^−1^) at the time of metabolic rate measurements were similar to those reported for zebra finches (mean±s.d. 5.694±1.09 mg kg^−1^; Gerson et al., 2019). This suggests that our results differ either as a result of a difference in treatment duration or from a higher resistance of song sparrows to the deleterious effects of MeHg on metabolic functions. Species with different diets and habitats may have evolved differently in their capacity to cope with environmental MeHg. Song sparrows feed primarily on aquatic-emergent invertebrates during the breeding season and may differ from granivorous zebra finches. Further comparative studies on this topic are warranted.

Similar to our study, a decrease in adult birds' BMR occurs under food restriction or food unpredictability in other species (Liang et al., 2015; Mckechnie and Lovegrove, 1999; Noakes et al., 2013), suggesting that in times of food scarcity, birds can adjust their energy expenditure. Such BMR adjustment can occur via a reduction of body temperature (Doucette et al., 2012; Graf et al., 1989; Hiebert, 1991; Moe et al., 2005), a change in behavioral activity (Dall and Witter, 1998; Pravosudov and Grubb, 1997) or a reduction in organ size (Moe et al., 2004, 2005). Such an energy conservation strategy during periods of hardship could allow the birds to allocate more energy towards other lengthy energetic processes such as growth and molt (compensation hypothesis; Moe et al., 2004; Welcker et al., 2015), although maybe not towards activity (e.g. migration or reproduction) as MMR and absolute metabolic scope were not affected. Interestingly, BMR reduction due to unpredictable food stress did not carry over into the post-exposure period as the October measure showed no treatment effects. The effect of food unpredictability was thus transient, possibly as a result of compensatory behavior of birds in terms of food consumption (reduced food consumption during the first 2 months of the experiment only; C. L. J. Bottini, B. Laxer, A. Khaira, B. A. Branfireun and S. A. MacDougall-Shackleton, unpublished) and/or the end of the treatment exposure. This finding is in agreement with prior studies where reduced BMR caused by food restriction during development returned to control levels after the stress period ended (Liang et al., 2015; O'Connor et al., 2000; Zubair and Leeson, 1994)*.*

Surprisingly, the influence of body mass on BMR was also transient as the scaling equation CI included zero in October but not in June–July. This could be due to the reduced sample size post-exposure and/or to the change in body composition in October. Indeed, the autumn increase in non-metabolically active tissue (fat mass) could have created too much variation to detect the influence of the reduction in lean mass (metabolically active tissue) on BMR. Because BMR is associated with survival and reproductive fitness (Burton et al., 2011; Jimeno et al., 2020; Rønning et al., 2016), BMR adjustment has ecological consequences for the birds.

In song sparrows, food restriction during early development had no long-term effects on MMR (Schmidt et al., 2012). However, food restriction did reduce MMR in domestic ducklings (*Anas platyrhyncos domesticus*) and European shag (*Phalacrocorax aristotelis*) nestlings (Moe et al., 2004, 2005). The lack of a food stress effect on MMR in song sparrows thus appears consistent across ages and differs from findings in other species. More studies are needed to better understand what factors affect song sparrow MMR values and how these may differ from other species.

In our study, stressed birds had close to significant higher factorial metabolic scope than unstressed birds due to the effect of the food stress-only treatment resulting in lower BMR than in control birds. Food restriction increased the factorial metabolic scope of ducklings (Moe et al., 2005). However, absolute metabolic scope was not affected by treatments (Fig. S2). These differences in results between absolute and fractional metabolic scope could be explained by the BMR results driving the factorial metabolic scope results (our study) while MMR is the primary driver of the absolute metabolic scope (Moe et al., 2005).

### Molt

Molt duration was longer in birds exposed to MeHg but not in those exposed to unpredictable food stress. The lack of effect of the unpredictable food stress treatment differs from prior studies showing reduced feather growth rate (Andrews et al., 2021; Murphy et al., 1988; Swaddle and Witter, 1997b). Because the food stress treatment had effects on other measures, this suggests that song sparrows could compensate feather growth despite unpredictable food stress. Furthermore, the sex effect with longer molt in females is to be taken with caution because of our low sample size of females at the end of the experiment (*n*=5) and would hence need to be confirmed by further studies.

The observed increase in molt duration due to MeHg differs from a prior study where molt rate was positively correlated with blood THg levels at molt onset in starlings (*Sturnus vulgaris*; Carlson et al., 2014). There are numerous methodological differences between that study and our experiment, including dose (0.19 versus 0.75 and 1.5 mg kg^−1^ in the starling study) and duration of MeHg exposure (3 months in our study versus 11 months in Carlson et al., 2014). In addition, the starlings were exposed to MeHg during the full duration of their molt. Hence, we cannot determine whether our differing results are caused by experimental differences or by the physiology of the studied species. Starlings have a longer molt (approximately 100 days; Carlson et al., 2014) than song sparrows (approximately 68 days: 9.69±1.67 weeks, mean±s.d., across all individuals in our experiment). Additionally, the starlings may not have been under the same migratory constraints as the song sparrows in this study, which could contribute to differing results. Further studies on molt speed under different challenging conditions are required.

Importantly, as molt timing is correlated with migration timing (Cristol et al., 2014; Pulido and Coppack, 2004; Stutchbury et al., 2011), a delayed molt duration could affect song sparrow migration departure. However, birds exposed to MeHg in this study had increased nocturnal migratory activity (Bottini et al., 2022), which was unexpected given their delayed molt. Alternatively, a molt–migration overlap, such as that observed in our study, could decrease the energy available for migratory behavior (Podlaszczuk et al., 2016) and negatively affect individual survival (Hemborg and Lundberg, 1998; Nolan et al., 1992).

### Feather quality

Contrary to prior studies (DesRochers et al., 2009; Murphy et al., 1988; Pap et al., 2008), we found weak support for an unpredictable food stress effect on feather length, mass and mass:length ratio. This suggests that the unpredictable food treatment was not associated with a strong stress response in song sparrows. However, our results did reveal an interaction between MeHg and primary feather number on feather mass:length ratio, feather mass (Table S3A) and feather length (Table S3B), with the interaction term being included within the top models. MeHg exposure resulted in increased mass:length ratio of P2, differing from the effect on other feathers. It is possible that only feathers growing during the exposure period were susceptible to MeHg effects. However, as P1 showed no strong effect of MeHg, more studies collecting multiple feathers are warranted. Prior studies documented MeHg effects on feather color and mass (Evers et al., 2008; Giraudeau et al., 2015; White and Cristol, 2014). As MeHg increased molt duration, and longer molt is associated with heavier feathers (De La Hera et al., 2010), the effects of MeHg on feather quality could be an indirect outcome of the MeHg effect on molt duration. In our study, the effect of MeHg on feather quality appears weak and it is difficult to determine whether it will have a positive or negative impact on feather durability or aerodynamics. More studies are hence warranted as a negative effect on feather quality could have strong consequences for the birds (Echeverry-Galvis and Hau, 2013; Møller and Nielsen, 2018; Swaddle et al., 1996).

Interestingly, we observed an increase in mass:length ratio from P1 to P9 feathers, also established in other species (Dawson, 2003, 2005). Dawson (2005) suggested that the outer primary feather's greater mass per length could be beneficial for the bird either as a protection against abrasion or for an aerodynamic purpose (Dawson, 2005).

We did not observe treatment effects on primary feather asymmetry. A prior study similarly did not find an effect of food deprivation on juvenile starlings' feather asymmetry (Swaddle and Witter, 1997b), but an effect was observed in adult females (Swaddle and Witter, 1994). An increased flight feather asymmetry was also observed in individuals exposed to high MeHg levels (Evers et al., 2008). Most of the molt in our study occurred during the post-exposure period, and we did not observe any strong effects of food stress or MeHg on feather quality and asymmetry, despite high blood THg levels at the end of exposure. This near null-effect suggests that prior exposure to MeHg would not affect feather quality if birds move out of contaminated areas prior to the onset of their molt. Hence, pre-molt movement and reduced foraging during molt (Brown and Bryant, 1996; Portugal et al., 2010; Rimmer, 1988) could be part of positively selected behaviors under contamination exposure.

### Conclusions

We measured the effects of unpredictable food stress and MeHg exposure on multiple measures of physiological performance associated with energy use. We observed that MeHg and food stress appear to act through different physiological mechanisms with no additive effect on body condition (fat and lean mass), metabolic rate, molt or feather quality. Our results suggest that wild songbirds may not be at risk of multiplicative effects from combined MeHg and food stress exposures on their energetic performance. However, MeHg effects on molt duration could potentially carry over across multiple annual cycle stages and may warrant further studies.

## Supplementary Material

10.1242/jexbio.246239_sup1 Supplementary information

## References

[JEB246239C1] Ackerman, J. T., Eagles-Smith, C. A., Herzog, M. P., Hartman, C. A., Peterson, S. H., Evers, D. C., Jackson, A. K., Elliott, J. E., Vander Pol, S. S. and Bryan, C. E. (2016). Avian mercury exposure and toxicological risk across western North America: a synthesis. *Sci. Total Environ.* 568, 749-769. 10.1016/j.scitotenv.2016.03.071 27093907 PMC5365029

[JEB246239C2] Acquarone, C., Cucco, M., Cauli, S. L. and Malacarne, G. (2002). Effects of food abundance and predictability on body condition and health parameters: experimental tests with the hooded crow. *Ibis* 144, E155-E163.

[JEB246239C3] Anderson, D. R. and Burnham, K. P. (2002). Avoiding pitfalls when using information-theoretic methods. *J. Wildl. Manage.* 66, 912. 10.2307/3803155

[JEB246239C4] Andrews, C., Zuidersma, E., Verhulst, S., Nettle, D. and Bateson, M. (2021). Exposure to food insecurity increases energy storage and reduces somatic maintenance in European starlings (*Sturnus vulgaris*). *R. Soc. Open Sci.* 8, 211099. 10.1098/rsos.211099 34540262 PMC8441118

[JEB246239C5] Arnold, P. A., Delean, S., Cassey, P. and White, C. R. (2021). Meta-analysis reveals that resting metabolic rate is not consistently related to fitness and performance in animals. *J. Comp. Physiol. B Biochem. Syst. Environ. Physiol.* 191, 1097-1110. 10.1007/s00360-021-01358-w 33721034

[JEB246239C6] Bateson, M., Andrews, C., Dunn, J., Egger, C. B. C. M., Gray, F., McHugh, M. and Nettle, D. (2021). Food insecurity increases energetic efficiency, not food consumption: an exploratory study in European starlings. *PeerJ* 9, e11541. 10.7717/peerj.11541 34123601 PMC8166238

[JEB246239C7] Bednekoff, P. A. and Krebs, J. R. (1995). Great tit fat reserves: effects of changing and unpredictable feeding day length. *Funct. Ecol.* 9, 457. 10.2307/2390009

[JEB246239C8] Bottini, C. L. J. (2022). Effects of methylmercury and unpredictable food stress exposure on songbirds’ physiology and seasonal transition. *PhD thesis*, The University of Western Ontario. https://ir.lib.uwo.ca/etd/9027/

[JEB246239C9] Bottini, C. L. J., MacDougall-Shackleton, S. A., Branfireun, B. A. and Hobson, K. A. (2021). Feathers accurately reflect blood mercury at time of feather growth in a songbird. *Sci. Total Environ.* 775, 145739. 10.1016/j.scitotenv.2021.145739 33621875

[JEB246239C10] Bottini, C. L. J., Whiley, R. E., Branfireun, B. A. and MacDougall-Shackleton, S. A. (2022). Effects of methylmercury and food stress on migratory activity in song sparrows, *Melospiza melodia*. *Horm. Behav.* 146, 105261. 10.1016/j.yhbeh.2022.105261 36126358

[JEB246239C11] Boyd, E. S., Yu, R.-Q., Barkay, T., Hamilton, T. L., Baxter, B. K., Naftz, D. L. and Marvin-DiPasquale, M. (2017). Effect of salinity on mercury methylating benthic microbes and their activities in Great Salt Lake, Utah. *Sci. Total Environ.* 581-582, 495-506. 10.1016/j.scitotenv.2016.12.157 28057343

[JEB246239C12] Brown, C. R. and Bryant, D. M. (1996). Energy expenditure during molt in dippers (*Cinclus cinclus*): no evidence of elevated costs. *Physiol. Zool.* 69, 1036-1056. 10.1086/physzool.69.5.30164245

[JEB246239C13] Brzȩk, P. and Konarzewski, M. (2001). Effect of food shortage on the physiology and competitive abilities of sand martin (Riparia riparia) nestlings. *J. Exp. Biol.* 204, 3065-3074. 10.1242/jeb.204.17.3065 11551994

[JEB246239C14] Burton, T., Killen, S. S., Armstrong, J. D. and Metcalfe, N. B. (2011). What causes intraspecific variation in resting metabolic rate and what are its ecological consequences? *Proc. R. Soc. B Biol. Sci.* 278, 3465-3473. 10.1098/rspb.2011.1778 PMC3189380 21957133

[JEB246239C15] Buttemer, W. A., Bauer, S., Emmenegger, T., Dimitrov, D., Peev, S. and Hahn, S. (2019). Moult-related reduction of aerobic scope in passerine birds. *J. Comp. Physiol. B Biochem. Syst. Environ. Physiol.* 189, 463-470. 10.1007/s00360-019-01213-z 30874899

[JEB246239C16] Carlson, J. R., Cristol, D. and Swaddle, J. P. (2014). Dietary mercury exposure causes decreased escape takeoff flight performance and increased molt rate in European starlings (*Sturnus vulgaris*). *Ecotoxicology* 23, 1464-1473. 10.1007/s10646-014-1288-5 25030113

[JEB246239C17] Carravieri, A., Vincze, O., Bustamante, P., Ackerman, J. T., Adams, E. M., Angelier, F., Chastel, O., Cherel, Y., Gilg, O., Golubova, E. et al. (2022). Quantitative meta–analysis reveals no association between mercury contamination and body condition in birds. *Biol. Rev.* 97, 1253-1271. 10.1111/brv.12840 35174617

[JEB246239C18] Chappell, M. A., Bech, C. and Buttemer, W. A. (1999). The relationship of central and peripheral organ masses to aerobic performance variation in house sparrows. *J. Exp. Biol.* 202, 2269-2279. 10.1242/jeb.202.17.2269 10441080

[JEB246239C19] Chételat, J., Ackerman, J. T., Eagles-Smith, C. A. and Hebert, C. E. (2020). Methylmercury exposure in wildlife: A review of the ecological and physiological processes affecting contaminant concentrations and their interpretation. *Sci. Total Environ.* 711, 135117. 10.1016/j.scitotenv.2019.135117 31831233

[JEB246239C20] Cornelius, E. A., Vézina, F., Regimbald, L., Hallot, F., Petit, M., Love, O. P. and Karasov, W. H. (2017). Chickadees faced with unpredictable food increase fat reserves but certain components of their immune function decline. *Physiol. Biochem. Zool.* 90, 190-200. 10.1086/689913 28277950

[JEB246239C21] Cristol, D. A., Brasso, R. L., Condon, A. M., Fovargue, R. E., Friedman, S. L., Hallinger, K. K., Monroe, A. P. and White, A. E. (2008). The movement of aquatic mercury through terrestrial food webs. *Science (80-.).* 320, 335-335. 10.1126/science.1154082 18420925

[JEB246239C22] Cristol, D. A., Johnson, K. M., Jenkins, K. D. and Hawley, D. M. (2014). Timing of feather molt related to date of spring migration in male White-throated sparrows, *Zonotrichia albicollis*. *J. Exp. Zool. Part A Ecol. Genet. Physiol.* 321, 586-594. 10.1002/jez.1899 25287905

[JEB246239C23] Cuthill, I. C., Maddocks, S. A., Weall, C. V. and Jones, E. K. M. (2000). Body mass regulation in response to changes in feeding predictability and overnight energy expenditure. *Behav. Ecol.* 11, 189-195. 10.1093/beheco/11.2.189

[JEB246239C24] Daan, S., Masman, D. and Groenewold, A. (1990). Avian basal metabolic rates: their association with body composition and energy expenditure in nature. *Am. J. Physiol. Regul. Integr. Comp. Physiol.* 259, 333-340. 10.1152/ajpregu.1990.259.2.R333 2386245

[JEB246239C25] Dall, S. R. X. and Witter, M. S. (1998). Feeding interruptions, diurnal mass changes and daily routines of behaviour in the zebra finch. *Anim. Behav.* 55, 715-725. 10.1006/anbe.1997.0749 9514680

[JEB246239C26] Danner, R. M., Greenberg, R. S., Danner, J. E. and Walters, J. R. (2015). Winter food limits timing of pre–alternate moult in a short–distance migratory bird. *Funct. Ecol.* 29, 259-267. 10.1111/1365-2435.12322

[JEB246239C27] Dawson, A. (2003). A detailed analysis of primary feather moult in the common starling *Sturnus vulgaris*- new feather mass increases at a constant rate. *Ibis* 145, E69-E76.

[JEB246239C28] Dawson, A. (2004). The effects of delaying the start of moult on the duration of moult, primary feather growth rates and feather mass in common starlings *Sturnus vulgaris*. *Ibis* 146, 493-500. 10.1111/j.1474-919x.2004.00290.x

[JEB246239C29] Dawson, A. (2005). The scaling of primary flight feather length and mass in relation to wing shape, function and habitat. *Ibis* 147, 283-292. 10.1111/j.1474-919x.2005.00400.x

[JEB246239C30] Dawson, A. (2018). Both low temperature and shorter duration of food availability delay testicular regression and affect the daily cycle in body temperature in a songbird. *Physiol. Biochem. Zool.* 91, 917-924. 10.1086/698109 29745775

[JEB246239C31] De La Hera, I., Pérez-Tris, J. and Tellería, J. L. (2010). Relationships among timing of moult, moult duration and feather mass in long-distance migratory passerines. *J. Avian Biol.* 41, 609-614. 10.1111/j.1600-048X.2010.05075.x

[JEB246239C32] DesRochers, D. W., Reed, J. M., Awerman, J., Kluge, J. A., Wilkinson, J., van Griethuijsen, L. I., Aman, J. and Romero, L. M. (2009). Exogenous and endogenous corticosterone alter feather quality. *Comp. Biochem. Physiol. A Mol. Integr. Physiol.* 152, 46-52. 10.1016/j.cbpa.2008.08.034 18804171

[JEB246239C139] Dietz, M. W., Piersma, T. and Dekinga, A. (1999). Body-building without power training: endogenously regulated pectoral muscle hypertrophy in confined shorebirds. *J. Exp. Biol.* 202, 2831-2837. 10504319 10.1242/jeb.202.20.2831

[JEB246239C33] Doucette, L. I., Brigham, R. M., Pavey, C. R. and Geiser, F. (2012). Prey availability affects daily torpor by free-ranging Australian owlet-nightjars (*Aegotheles cristatus*). *Oecologia* 169, 361-372. 10.1007/s00442-011-2214-7 22173484

[JEB246239C34] Dunn, O. J. (1964). Multiple comparisons using rank sums. *Technometrics* 6, 241-252. 10.1080/00401706.1964.10490181

[JEB246239C35] Echeverry-Galvis, M. A. and Hau, M. (2013). Flight performance and feather quality: paying the price of overlapping moult and breeding in a tropical highland bird. *PLoS One* 8, e61106. 10.1371/journal.pone.0061106 23667431 PMC3648541

[JEB246239C36] U.S. EPA (1998). *Method 7473 (SW-846): Mercury in Solids and Solutions by Thermal Decomposition, Amalgamation, and Atomic Absorption Spectrophotometry*. Washington, DC: U.S. EPA.

[JEB246239C37] Evers, D. C., Savoy, L. J., DeSorbo, C. R., Yates, D. E., Hanson, W., Taylor, K. M., Siegel, L. S., Cooley, J. H., Bank, M. S., Major, A. et al. (2008). Adverse effects from environmental mercury loads on breeding common loons. *Ecotoxicology* 17, 69-81. 10.1007/s10646-007-0168-7 17909967

[JEB246239C38] Fokidis, H. B., Des Roziers, M. B., Sparr, R., Rogowski, C., Sweazea, K. and Deviche, P. (2012). Unpredictable food availability induces metabolic and hormonal changes independent of food intake in a sedentary songbird. *J. Exp. Biol.* 215, 2920-2930. 10.1242/jeb.071043 22837467

[JEB246239C39] Fox, A. D. and Kahlert, J. (2005). Changes in body mass and organ size during wing moult in non-breeding greylag geese *Anser anser*. *J. Avian Biol.* 36, 538-548. 10.1111/j.0908-8857.2005.03301.x

[JEB246239C40] Fox, A. D., Flint, P. L., Hohman, W. L. and Savard, J. P. L. (2014). Waterfowl habitat use and selection during the remigial moult period in the northern hemisphere. *Wildfowl* Special Issue No. 4, 131-168.

[JEB246239C41] Gerson, A. R. and Guglielmo, C. G. (2011). Flight at low ambient humidity increases protein catabolism in migratory birds. *Science* 333, 1434-1436. 10.1126/science.1210449 21903811

[JEB246239C42] Gerson, A. R., Cristol, D. A. and Seewagen, C. L. (2019). Environmentally relevant methylmercury exposure reduces the metabolic scope of a model songbird. *Environ. Pollut.* 246, 790-796. 10.1016/j.envpol.2018.12.072 30623835

[JEB246239C43] Giraudeau, M., Mateos-Gonzalez, F., Cotín, J., Pagani-Nuñez, E., Torné-Noguera, A. and Senar, J. C. (2015). Metal exposure influences the melanin and carotenoid-based colorations in great tits. *Sci. Total Environ.* 532, 512-516. 10.1016/j.scitotenv.2015.06.021 26100730

[JEB246239C44] Goodchild, C. G., Simpson, A. M., Minghetti, M. and DuRant, S. E. (2019). Bioenergetics-adverse outcome pathway: linking organismal and suborganismal energetic endpoints to adverse outcomes. *Environ. Toxicol. Chem.* 38, 27-45. 10.1002/etc.4280 30259559

[JEB246239C45] Graf, R., Krishna, S. and Heller, H. C. (1989). Regulated nocturnal hypothermia induced in pigeons by food deprivation. *Am. J. Physiol. Regul. Integr. Comp. Physiol.* 256, R733-R738. 10.1152/ajpregu.1989.256.3.R733 2923260

[JEB246239C46] Grieves, L. A., Bernards, M. A. and MacDougall-Shackleton, E. A. (2019a). Behavioural responses of songbirds to preen oil odour cues of sex and species. *Anim. Behav.* 156, 57-65. 10.1016/j.anbehav.2019.06.035

[JEB246239C47] Grieves, L. A., Gloor, G. B., Bernards, M. A. and MacDougall-Shackleton, E. A. (2019b). Songbirds show odour-based discrimination of similarity and diversity at the major histocompatibility complex. *Anim. Behav.* 158, 131-138. 10.1016/j.anbehav.2019.10.005

[JEB246239C48] Grieves, L. A., Bottini, C. L. J., Branfireun, B. A., Bernards, M. A., MacDougall-Shackleton, S. A. and MacDougall-Shackleton, E. A. (2020). Food stress, but not experimental exposure to mercury, affects songbird preen oil composition. *Ecotoxicology* 29, 275-285. 10.1007/s10646-020-02171-x 32036507

[JEB246239C49] Guglielmo, C. G., McGuire, L. P., Gerson, A. R. and Seewagen, C. L. (2011). Simple, rapid, and non-invasive measurement of fat, lean, and total water masses of live birds using quantitative magnetic resonance. *J. Ornithol.* 152, 75-85. 10.1007/s10336-011-0724-z

[JEB246239C50] Guillemette, M., Pelletier, D., Grandbois, J. M. and Butler, P. J. (2007). Flightlessness and the energetic cost of wing molt in a large sea duck. *Ecology* 88, 2936-2945. 10.1890/06-1751.1 18051662

[JEB246239C51] Harding, K. M., Gowland, J. A. and Dillon, P. J. (2006). Mercury concentration in black flies *Simulium* spp. (*Diptera, Simuliidae*) from soft-water streams in Ontario, Canada. *Environ. Pollut.* 143, 529-535. 10.1016/j.envpol.2005.11.040 16490293

[JEB246239C52] Harrison, X. A., Blount, J. D., Inger, R., Norris, D. R. and Bearhop, S. (2011). Carry-over effects as drivers of fitness differences in animals. *J. Anim. Ecol.* 80, 4-18. 10.1111/j.1365-2656.2010.01740.x 20726924

[JEB246239C53] Heddle, C., Elliott, J. E., Brown, T. M., Eng, M. L., Perkins, M., Basu, N. and Williams, T. D. (2020). Continuous exposure to mercury during embryogenesis and chick development affects later survival and reproduction of zebra finch (*Taeniopygia guttata*). *Ecotoxicology* 29, 1117-1127. 10.1007/s10646-019-02074-6 31352572

[JEB246239C54] Hemborg, C. and Lundberg, A. (1998). Costs of overlapping reproduction and moult in passerine birds: an experiment with the pied flycatcher. *Behav. Ecol. Sociobiol.* 43, 19-23. 10.1007/s002650050462

[JEB246239C55] Henny, C. J., Hill, E. F., Hoffman, D. J., Spalding, M. G. and Grove, R. A. (2002). Nineteenth century mercury: hazard to wading birds and cormorants of the Carson River, Nevada. *Ecotoxicology* 11, 213-231. 10.1023/A:1016327602656 12211695

[JEB246239C56] Hiebert, S. M. (1991). Seasonal differences in the response of rufous hummingbirds to food restriction: body mass and the use of torpor. *Condor* 93, 526-537. 10.2307/1368184

[JEB246239C57] Hoffman, D. J., Henny, C. J., Hill, E. F., Grove, R. A., Kaiser, J. L. and Stebbins, K. R. (2009). Mercury and drought along the lower Carson River, Nevada: III. Effects on blood and organ biochemistry and histopathology of snowy egrets and black-crowned night-herons on Lahontan Reservoir, 2002-2006. *J. Toxicol. Environ. Heal. Part A Curr. Issues* 72, 1223-1241. 10.1080/15287390903129218 20077191

[JEB246239C58] Husak, J. F. and Lailvaux, S. P. (2017). How do we measure the cost of whole-organism performance traits? *Integr. Comp. Biol.* 57, 333-343. 10.1093/icb/icx048 28859402

[JEB246239C59] Jimeno, B., Prichard, M. R., Landry, D., Wolf, C., Larkin, B., Cheviron, Z. and Breuner, C. (2020). Metabolic rates predict baseline corticosterone and reproductive output in a free-living passerine. *Integr. Org. Biol.* 2, obaa030. 10.1093/iob/obaa030 33791569 PMC7794023

[JEB246239C60] Jones, T. M. and Ward, M. P. (2020). Pre- to post-fledging carryover effects and the adaptive significance of variation in wing development for juvenile songbirds. *J. Anim. Ecol.* 89, 2235-2245. 10.1111/1365-2656.13285 32596836

[JEB246239C61] Jonsson, S., Andersson, A., Nilsson, M. B., Skyllberg, U., Lundberg, E., Schaefer, J. K., Åkerblom, S. and Björn, E. (2017). Terrestrial discharges mediate trophic shifts and enhance methylmercury accumulation in estuarine biota. *Sci. Adv.* 3, e1601239. 10.1126/sciadv.1601239 28138547 PMC5271591

[JEB246239C62] Kiat, Y., Izhaki, I. and Sapir, N. (2019). The effects of long-distance migration on the evolution of moult strategies in Western-Palearctic passerines. *Biol. Rev.* 94, 700-720. 10.1111/brv.12474 30334341

[JEB246239C63] Konarzewski, M. and Ksiazek, A. (2013). Determinants of intra-specific variation in basal metabolic rate. *J. Comp. Physiol. B Biochem. Syst. Environ. Physiol.* 183, 27-41. 10.1007/s00360-012-0698-z PMC3536993 22847501

[JEB246239C64] Koren, L., Nakagawa, S., Burke, T., Soma, K. K., Wynne-Edwards, K. E. and Geffen, E. (2012). Non-breeding feather concentrations of testosterone, corticosterone and cortisol are associated with subsequent survival in wild house sparrows. *Proc. R. Soc. B Biol. Sci.* 279, 1560-1566. 10.1098/rspb.2011.2062 PMC3282351 22090380

[JEB246239C65] Krabbenhoft, D. P. and Sunderland, E. M. (2013). Global change and mercury. *Science* 341, 1457-1458. 10.1126/science.1242838 24072910

[JEB246239C66] Kuznetsova, A., Brockhoff, P. and Christensen, R. (2017). lmerTest package: tests in linear mixed effects models. *J. Stat. Softw.* 82, 1-26. 10.18637/jss.v082.i13

[JEB246239C67] Latta, S. C., Cabezas, S., Mejia, D. A., Paulino, M. M., Almonte, H., Miller-Butterworth, C. M. and Bortolotti, G. R. (2016). Carry-over effects provide linkages across the annual cycle of a Neotropical migratory bird, the Louisiana waterthrush *Parkesia motacilla*. *Ibis* 158, 395-406. 10.1111/ibi.12344

[JEB246239C68] Liang, Q. J., Zhao, L., Wang, J. Q., Chen, Q., Zheng, W. H. and Liu, J. S. (2015). Effect of food restriction on the energy metabolism of the Chinese bulbul (*Pycnonotus sinensis*). *Dongwuxue. Yanjiu.* 36, 79-87. 10.13918/j.issn.2095-8137.2015.2.79 25855226 PMC4790253

[JEB246239C69] Lighton, J. R. (2008). *Measuring Metabolic Rates: a Manual for Scientists*. Oxford University Press.

[JEB246239C70] Lindström, Å., Visser, G. H. and Daan, S. (1993). The energetic cost of feather synthesis is proportional to basal metabolic rate. *Physiol. Zool.* 66, 490-510. 10.1086/physzool.66.4.30163805

[JEB246239C71] Lupi, S., Goymann, W., Cardinale, M. and Fusani, L. (2016). Physiological conditions influence stopover behavior of short-distance migratory passerines. *J. Ornithol.* 157, 583-589. 10.1007/s10336-015-1303-5

[JEB246239C72] Lustick, S. (1970). Energy requirements of molt in cowbirds. *Auk* 87, 742-746. 10.2307/4083708

[JEB246239C73] Ma, Y., Branfireun, B. A., Hobson, K. A. and Guglielmo, C. G. (2018). Evidence of negative seasonal carry-over effects of breeding ground mercury exposure on survival of migratory songbirds. *J. Avian Biol.* 49, 1-8.

[JEB246239C74] Mahbub, K. R., Krishnan, K., Naidu, R., Andrews, S. and Megharaj, M. (2017). Mercury toxicity to terrestrial biota. *Ecol. Indic.* 74, 451-462. 10.1016/j.ecolind.2016.12.004

[JEB246239C75] Marmillot, V., Gauthier, G., Cadieux, M. C. and Legagneux, P. (2016). Plasticity in moult speed and timing in an arctic-nesting goose species. *J. Avian Biol.* 47, 650-658. 10.1111/jav.00982

[JEB246239C76] Marzal, A., Asghar, M., Rodríguez, L., Reviriego, M., Hermosell, I. G., Balbontín, J., Garcia-Longoria, L., de Lope, F. and Bensch, S. (2013). Co-infections by malaria parasites decrease feather growth but not feather quality in house martin. *J. Avian Biol.* 44, 437-444. 10.1111/j.1600-048X.2013.00178.x

[JEB246239C77] Mathot, K. J., Kok, E. M. A., Burant, J. B., Dekinga, A., Manche, P., Saintonge, D. and Piersma, T. (2019). Evolutionary design of a flexible, seasonally migratory, avian phenotype: why trade gizzard mass against pectoral muscle mass? *Proc. R. Soc. B Biol. Sci.* 286, 20190518. 10.1098/rspb.2019.0518 PMC6545091 31113330

[JEB246239C78] McKechnie, A. E. and Lovegrove, B. G. (1999). Circadian metabolic responses to food deprivation in the black-shouldered kite. *Condor* 101, 426-432. 10.2307/1370010

[JEB246239C79] McKechnie, A. E., Noakes, M. J. and Smit, B. (2015). Global patterns of seasonal acclimatization in avian resting metabolic rates. *J. Ornithol.* 156, 367-376. 10.1007/s10336-015-1186-5

[JEB246239C80] McNab, B. K. (1997). On the utility of uniformity in the definition of basal rate of metabolism. *Physiol. Zool.* 70, 718-720. 10.1086/515881 9361146

[JEB246239C81] Merritt, K. A. and Amirbahman, A. (2009). Mercury methylation dynamics in estuarine and coastal marine environments: a critical review. *Earth-Sci. Rev.* 96, 54-66. 10.1016/j.earscirev.2009.06.002

[JEB246239C82] Moe, B., Brunvoll, S., Mork, D., Brobakk, T. E. and Bech, C. (2004). Developmental plasticity of physiology and morphology in diet-restricted European shag nestlings (*Phalacrocorax aristotelis*). *J. Exp. Biol.* 207, 4067-4076. 10.1242/jeb.01226 15498952

[JEB246239C83] Moe, B., Stølevik, E. and Bech, C. (2005). Ducklings exhibit substantial energy-saving mechanisms as a response to short-term food shortage. *Physiol. Biochem. Zool.* 78, 90-104. 10.1086/425199 15702467

[JEB246239C84] Møller, A. P. and Nielsen, J. T. (2018). The trade-off between rapid feather growth and impaired feather quality increases risk of predation. *J. Ornithol.* 159, 165-171. 10.1007/s10336-017-1483-2

[JEB246239C85] Moore, M. P. and Martin, R. A. (2019). On the evolution of carry-over effects. *J. Anim. Ecol.* 88, 1832-1844. 10.1111/1365-2656.13081 31402447

[JEB246239C86] Munthe, J., Bodaly, R. A. D., Branfireun, B. A., Driscoll, C. T., Gilmour, C. C., Harris, R., Horvat, M., Lucotte, M. and Malm, O. (2007). Recovery of mercury-contaminated fisheries. *AMBIO A J. Hum. Environ.* 36, 33-44. 10.1579/0044-7447(2007)36[33:ROMF]2.0.CO;2 17408189

[JEB246239C87] Murphy, M. E. and King, J. R. (1992). Energy and nutrient use during moult by white-crowned sparrows *Zonotrichia leucophrys gambelii*. *Ornis Scand.* 23, 304. 10.2307/3676654

[JEB246239C88] Murphy, M. E. and Taruscio, T. G. (1995). Sparrows increase their rates of tissue and whole-body protein synthesis during the annual molt. *Comp. Biochem. Physiol. Part A Physiol.* 111, 385-396. 10.1016/0300-9629(95)00039-A

[JEB246239C89] Murphy, M. E., King, J. R. and Lu, J. (1988). Malnutrition during the postnuptial molt of white-crowned sparrows: feather growth and quality. *Can. J. Zool.* 66, 1403-1413. 10.1139/z88-206

[JEB246239C90] Newman, M. C., Xu, X., Condon, A. and Liang, L. (2011). Floodplain methylmercury biomagnification factor higher than that of the contiguous river (South River, Virginia USA). *Environ. Pollut.* 159, 2840-2844. 10.1016/j.envpol.2011.04.045 21621888

[JEB246239C91] Noakes, M. J., Smit, B., Wolf, B. O. and McKechnie, A. E. (2013). Thermoregulation in African green pigeons (*Treron calvus*) and a re-analysis of insular effects on basal metabolic rate and heterothermy in columbid birds. *J. Comp. Physiol. B Biochem. Syst. Environ. Physiol.* 183, 969-982. 10.1007/s00360-013-0763-2 23689380

[JEB246239C92] Nolan, V., Ketterson, E. D., Ziegenfus, C., Cullen, D. P. and Chandler, C. R. (1992). Testosterone and avian life histories: effects of experimentally elevated testosterone on prebasic molt and survival in male dark-eyed juncos. *Condor* 94, 364-370. 10.2307/1369209

[JEB246239C93] O'Connor, K. I., Taylor, A. C. and Metcalfe, N. B. (2000). The stability of standard metabolic rate during a period of food deprivation in juvenile Atlantic salmon. *J. Fish Biol.* 57, 41-51. 10.1111/j.1095-8649.2000.tb00774.x

[JEB246239C94] O'Connor, C. M., Norris, D. R., Crossin, G. T. and Cooke, S. J. (2014). Biological carryover effects: linking common concepts and mechanisms in ecology and evolution. *Ecosphere* 5, 1-11. 10.1890/ES13-00388.1

[JEB246239C95] Pap, P. L., Vágási, C. I., Czirják, G. Á. and Barta, Z. (2008). Diet quality affects postnuptial molting and feather quality of the house sparrow (*Passer domesticus*): interaction with humoral immune function? *Can. J. Zool.* 86, 834-842. 10.1139/Z08-060

[JEB246239C96] Paris, O. J., Swaddle, J. P. and Cristol, D. A. (2018). Exposure to dietary methyl-mercury solely during embryonic and juvenile development halves subsequent reproductive success in adult zebra finches. *Environ. Sci. Technol.* 52, 3117-3124. 10.1021/acs.est.7b04752 29350925

[JEB246239C97] Petit, M., Lewden, A. and Vézina, F. (2014). How does flexibility in body composition relate to seasonal changes in metabolic performance in a small passerine wintering at northern latitude? *Physiol. Biochem. Zool.* 87, 539-549. 10.1086/676669 24940918

[JEB246239C98] Pettersen, A. K., Marshall, D. J. and White, C. R. (2018). Understanding variation in metabolic rate. *J. Exp. Biol.* 221, jeb166876. 10.1242/jeb.166876 29326115

[JEB246239C99] Pierce, B. J., McWilliams, S. R., O'Connor, T. P., Place, A. R. and Guglielmo, C. G. (2005). Effect of dietary fatty acid composition on depot fat and exercise performance in a migrating songbird, the red-eyed vireo. *J. Exp. Biol.* 208, 1277-1285. 10.1242/jeb.01493 15781888

[JEB246239C100] Podlaszczuk, P., Kamiński, M., Włodarczyk, R., Kaczmarek, K., Janiszewski, T. and Minias, P. (2016). Plumage quality mediates a life-history trade-off in a migratory bird. *Front. Zool.* 13, 47. 10.1186/s12983-016-0179-4 27766111 PMC5057445

[JEB246239C101] Portugal, S. J., Green, J. A. and Butler, P. J. (2007). Annual changes in body mass and resting metabolism in captive barnacle geese (*Branta leucopsis*): the importance of wing moult. *J. Exp. Biol.* 210, 1391-1397. 10.1242/jeb.004598 17401121

[JEB246239C102] Portugal, S. J., Isaac, R., Quinton, K. L. and Reynolds, S. J. (2010). Do captive waterfowl alter their behaviour patterns during their flightless period of moult? *J. Ornithol.* 151, 443-448. 10.1007/s10336-009-0474-3

[JEB246239C103] Pravosudov, V. V. and Grubb, T. C. (1997). Management of fat reserves and food caches in tufted titmice (*Parus bicolor*) in relation to unpredictable food supply. *Behav. Ecol.* 8, 332-339. 10.1093/beheco/8.3.332

[JEB246239C104] Price, E. R. and Guglielmo, C. G. (2009). The effect of muscle phospholipid fatty acid composition on exercise performance: a direct test in the migratory white-throated sparrow (*Zonotrichia albicollis*). *Am. J. Physiol. Regul. Integr. Comp. Physiol.* 297, 775-782. 10.1152/ajpregu.00150.2009 19587112

[JEB246239C105] Pulido, F. and Coppack, T. (2004). Correlation between timing of juvenile moult and onset of migration in the blackcap, *Sylvia atricapilla*. *Anim. Behav.* 68, 167-173. 10.1016/j.anbehav.2003.11.006

[JEB246239C106] Rimmer, C. C. (1988). Timing of the definitive prebasic molt in yellow warblers at James Bay, Ontario. *Condor* 90, 141-156. 10.2307/1368443

[JEB246239C107] Rimmer, C. C., Mcfarland, K. P., Evers, D. C., Miller, E. K., Aubry, Y., Busby, D. and Taylor, R. J. (2005). Mercury concentrations in Bicknell's thrush and other insectivorous passerines in Montane forests of northeastern North America. *Ecotoxicology* 14, 223-240. 10.1007/s10646-004-6270-1 15931968

[JEB246239C108] Romero, L. M., Strochlic, D. and Wingfield, J. C. (2005). Corticosterone inhibits feather growth: potential mechanism explaining seasonal down regulation of corticosterone during molt. *Comp. Biochem. Physiol. A Mol. Integr. Physiol.* 142, 65-73. 10.1016/j.cbpa.2005.07.014 16125989

[JEB246239C109] Rønning, B., Mortensen, A. S., Moe, B., Chastel, O., Arukwe, A. and Bech, C. (2009). Food restriction in young Japanese quails: effects on growth, metabolism, plasma thyroid hormones and mRNA species in the thyroid hormone signalling pathway. *J. Exp. Biol.* 212, 3060-3067. 10.1242/jeb.029835 19749098

[JEB246239C110] Rønning, B., Broggi, J., Bech, C., Moe, B., Ringsby, T. H., Pärn, H., Hagen, I. J., Sæther, B. E. and Jensen, H. (2016). Is basal metabolic rate associated with recruit production and survival in free-living house sparrows? *Funct. Ecol.* 30, 1140-1148. 10.1111/1365-2435.12597

[JEB246239C111] Root, T. L., O'Connor, T. P. and Dawson, W. R. (1991). Standard metabolic level and insulative characteristics of eastern house finches, *Carpodacus mexicanus* (Muller). *Physiol. Zool.* 64, 1279-1295. 10.1086/physzool.64.5.30156245

[JEB246239C112] Schaefer, K., Elshorbany, Y., Jafarov, E., Schuster, P. F., Striegl, R. G., Wickland, K. P. and Sunderland, E. M. (2020). Potential impacts of mercury released from thawing permafrost. *Nat. Commun.* 11, 4650. 10.1038/s41467-020-18398-5 32938932 PMC7494925

[JEB246239C113] Schaub, M., Jenni, L. and Bairlein, F. (2008). Fuel stores, fuel accumulation, and the decision to depart from a migration stopover site. *Behav. Ecol.* 19, 657-666. 10.1093/beheco/arn023

[JEB246239C114] Scheiman, D. M. and Dunning, J. B. (2004). A case of arrested molt in the bobolink. *North Am. Bird Bander* 29, 105-107.

[JEB246239C115] Schmaljohann, H. and Naef-Daenzer, B. (2011). Body condition and wind support initiate the shift of migratory direction and timing of nocturnal departure in a songbird. *J. Anim. Ecol.* 80, 1115-1122. 10.1111/j.1365-2656.2011.01867.x 21615404

[JEB246239C116] Schmidt, K. L., MacDougall-Shackleton, E. A. and MacDougall-Shackleton, S. A. (2012). Developmental stress has sex-specific effects on nestling growth and adult metabolic rates but no effect on adult body size or body composition in song sparrows. *J. Exp. Biol.* 215, 3207-3217. 10.1242/jeb.068965 22693025

[JEB246239C117] Schoenle, L. A., Zimmer, C. and Vitousek, M. N. (2018). Understanding context dependence in glucocorticoid–fitness relationships: the role of the nature of the challenge, the intensity and frequency of stressors, and life history. *Integr. Comp. Biol.* 58, 777-789. 10.1093/icb/icy046 29889246

[JEB246239C118] Scoville, S. A., Varian-Ramos, C. W., Adkins, G. A., Swaddle, J. P., Saha, M. S. and Cristol, D. A. (2020). Mercury delays cerebellar development in a model songbird species, the zebra finch. *Ecotoxicology* 29, 1128-1137. 10.1007/s10646-020-02270-9 32827288

[JEB246239C119] Slagsvold, T. and Dale, S. (1996). Disappearance of female pied flycatchers in relation to breeding stage and experimentally induced molt. *Ecology* 77, 461-471. 10.2307/2265622

[JEB246239C120] Stutchbury, B. J. M., Gow, E. A., Done, T., MacPherson, M., Fox, J. W. and Afanasyev, V. (2011). Effects of post-breeding moult and energetic condition on timing of songbird migration into the tropics. *Proc. Biol. Sci.* 278, 131-137. 10.1098/rspb.2010.1220 20659932 PMC2992728

[JEB246239C121] Swaddle, J. P. and Witter, M. S. (1994). Food, feathers and fluctuating asymmetries. *Proc. R. Soc. B Biol. Sci.* 255, 147-152. 10.1098/rspb.1994.0021

[JEB246239C122] Swaddle, J. P. and Witter, M. S. (1997a). The effects of molt on the flight performance, body mass and behavior of European starlings. *Can. J. Zool.* 75, 1135-1146. 10.1139/z97-136

[JEB246239C123] Swaddle, J. P. and Witter, M. S. (1997b). Food availability and primary feather molt in European starlings, *Sturnus vulgaris*. *Can. J. Zool.* 75, 948-953. 10.1139/z97-114

[JEB246239C124] Swaddle, J. P., Witter, M. S., Cuthill, I. C., Budden, A. and McCowen, P. (1996). Plumage condition affects flight performance in common starlings. *J. Avian Biol.* 27, 103-111. 10.2307/3677139

[JEB246239C125] Swanson, D. L. (2010). Seasonal metabolic variation in birds: functional and mechanistic correlates. In: *Current Ornithology*, pp. 75-129. New York, NY: Springer.

[JEB246239C126] Swanson, D. L., McKechnie, A. E. and Vézina, F. (2017). How low can you go? An adaptive energetic framework for interpreting basal metabolic rate variation in endotherms. *J. Comp. Physiol. B Biochem. Syst. Environ. Physiol.* 187, 1039-1056. 10.1007/s00360-017-1096-3 28401293

[JEB246239C127] Thompson, D. R. and Furness, R. W. (1989). The chemical form of mercury stored in South Atlantic seabirds. *Environ. Pollut.* 60, 305-317. 10.1016/0269-7491(89)90111-5 15092383

[JEB246239C128] Vallverdú-Coll, N., Ortiz-Santaliestra, M. E., Mougeot, F., Vidal, D. and Mateo, R. (2015). Sublethal Pb exposure produces season-dependent effects on immune response, oxidative balance and investment in carotenoid-based coloration in red-legged partridges. *Environ. Sci. Technol.* 49, 3839-3850. 10.1021/es505148d 25674808

[JEB246239C129] Vaucoulon, P., Groscolas, R. and Barre, H. (1985). Photoperiodic and food control of moult in the juvenile king penguin (*Aptenodytes patagonicus*). *Comp. Biochem. Physiol. A Physiol.* 81, 347-351. 10.1016/0300-9629(85)90146-X

[JEB246239C130] Vézina, F., Gustowska, A., Jalvingh, K. M., Chastel, O. and Piersma, T. (2009). Hormonal correlates and thermoregulatory consequences of molting on metabolic rate in a northerly wintering shorebird. *Physiol. Biochem. Zool.* 82, 129-142. 10.1086/596512 19199554

[JEB246239C131] Weber, J. H. (1993). Review of possible paths for abiotic methylation of mercury(II) in the aquatic environment. *Chemosphere* 26, 2063-2077. 10.1016/0045-6535(93)90032-Z

[JEB246239C132] Welcker, J., Speakman, J. R., Elliott, K. H., Hatch, S. A. and Kitaysky, A. S. (2015). Resting and daily energy expenditures during reproduction are adjusted in opposite directions in free–living birds. *Funct. Ecol.* 29, 250-258. 10.1111/1365-2435.12321

[JEB246239C133] White, A. E. and Cristol, D. A. (2014). Plumage coloration in belted kingfishers (*Megaceryle alcyon*) at a mercury-contaminated river. *Waterbirds* 37, 144-152. 10.1675/063.037.0203

[JEB246239C134] White, C. R. and Kearney, M. R. (2013). Determinants of inter-specific variation in basal metabolic rate. *J. Comp. Physiol. B Biochem. Syst. Environ. Physiol.* 183, 1-26. 10.1007/s00360-012-0676-5 23001691

[JEB246239C135] Williams, T. D. (2018). Physiology, activity and costs of parental care in birds. *J. Exp. Biol.* 221, jeb169433. 10.1242/jeb.169433 30201656

[JEB246239C136] Witter, M. S., Swaddle, J. P. and Cuthill, I. C. (1995). Periodic food availability and strategic regulation of body mass in the European starling, *Sturnus vulgaris*. *Funct. Ecol.* 9, 568-574. 10.2307/2390146

[JEB246239C137] Zhang, Y., Yang, K., Yang, P., Su, Y., Zheng, W. and Liu, J. (2018). Food restriction decreases BMR, body and organ mass, and cellular energetics, in the Chinese bulbul (*Pycnonotus sinensis*). *Avian Res.* 9, 39. 10.1186/s40657-018-0131-8

[JEB246239C138] Zubair, A. K. and Leeson, S. (1994). Effect of early feed restriction and realimentation on heat production and changes in sizes of digestive organs of male broilers. *Poult. Sci.* 73, 529-538. 10.3382/ps.0730529 8202432

