# Supplementary material for: Effects of sublethal methylmercury and food stress on songbird energetic performance: metabolic rates, molt and feather quality
Source: J Exp Biol. 2024 Jul 5;227(13):jeb246239. doi: 10.1242/jeb.246239 (PMC11418191; doi:10.1242/jeb.246239)
Supplement: Supplementary information [file jexbio-227-246239-s1.pdf]

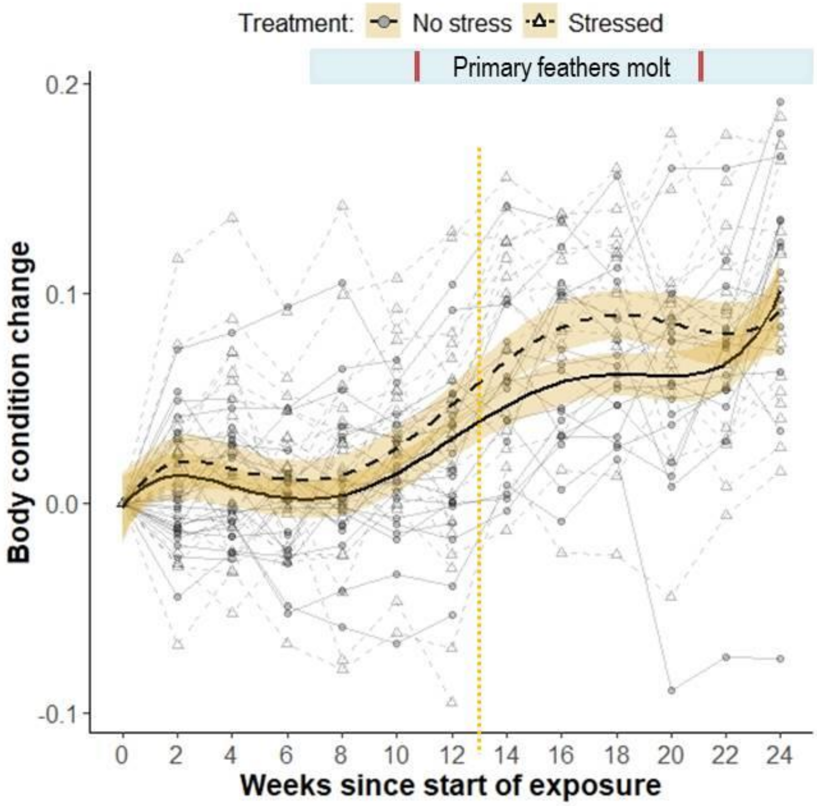

Fig. S1. Change in body condition (mean ± SE of mass / tarsus length) according to time (weeks) and food stress treatment (pooled across MeHg treatments). Time-point 0 corresponds to the pre-experiment body condition measured on 14-15 May. The yellow dotted vertical line indicates the end of treatment exposure and euthanasia of 2-4 birds per treatment. The horizontal blue bar indicates the start and end of the molt period, with vertical red lines indicating the mean date of molt start (3 August) and molt end (12 October). Regression lines were fitted via a linear model with a polynomial function of 5 degrees. The solid line and filled circles (n = 16 by the end of experiment) indicate birds with no food stress (MeHg-only and control treatment), while dashed line and open triangles (n = 16) indicate birds with food stress (stress-only and combined MeHg and stress treatments). Connected symbols represent data for individual birds.

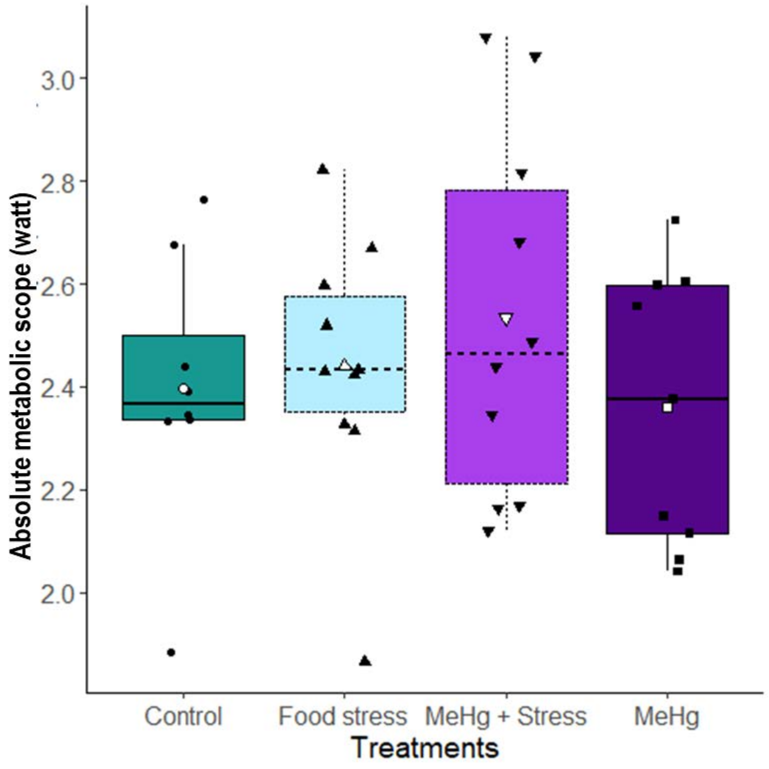

Fig. S2. Variation of absolute metabolic scope (MMR-BMR in W) according to treatment groups. Boxplots indicate 25th, 50th, and 75th percentiles and whiskers indicate range, with individual jittered data points overlaid. Boxplots colour and symbols indicate treatment groups: control (dark blue with circles), food stress (light blue with triangles), combined MeHg and food stress (light purple with inverted triangles), MeHg (dark purple with squares). The white symbols indicate the mean absolute metabolic scope for each treatment group.

The saturated linear model independent variables included the interaction of stress and mercury, the effect of sex, and mass when exiting the MMR metabolic chamber. We followed the protocol described in the main manuscript to perform the candidate model selection and results extraction. Four candidate models had a ΔAICc lower than two. The best candidate model included the effect of mass and sex only (df = 4; logLik = 2.041; AICc = 5.13; ΔAICc = 0; weight = 0.35), the second best included only the effect of mass (df = 3; logLik = 0.58; AICc = 5.55; ΔAICc = 0.42; weight = 0.28), the third model included the effect of mass, sex and stress (df = 5; logLik = 2.74; AICc = 6.39; ΔAICc = 1.26; weight = 0.19) and the fourth included the effect of mass and stress exposure (df = 4; logLik = 1.38; AICc = 6.46; ΔAICc = 1.33; weight = 0.18). The absolute metabolic scope increased with bird's mass when exiting MMR (RI = 1; Estimate = 0.12; SE = 0.043; 95% CI = 0.030 to 0.20; p = 0.0082), while sex (RI = 0.54; Estimate = 0.19; SE = 0.12; 95% CI = -0.045 to 0.43; p = 0.11) and stress exposure (RI = 0.37; Estimate = 0.092; SE = 0.079; 95% CI = -0.067 to 0.25; p = 0.26) had no or weak effect on the bird's metabolic scope.

Journal of Experimental Biology • Supplementary information

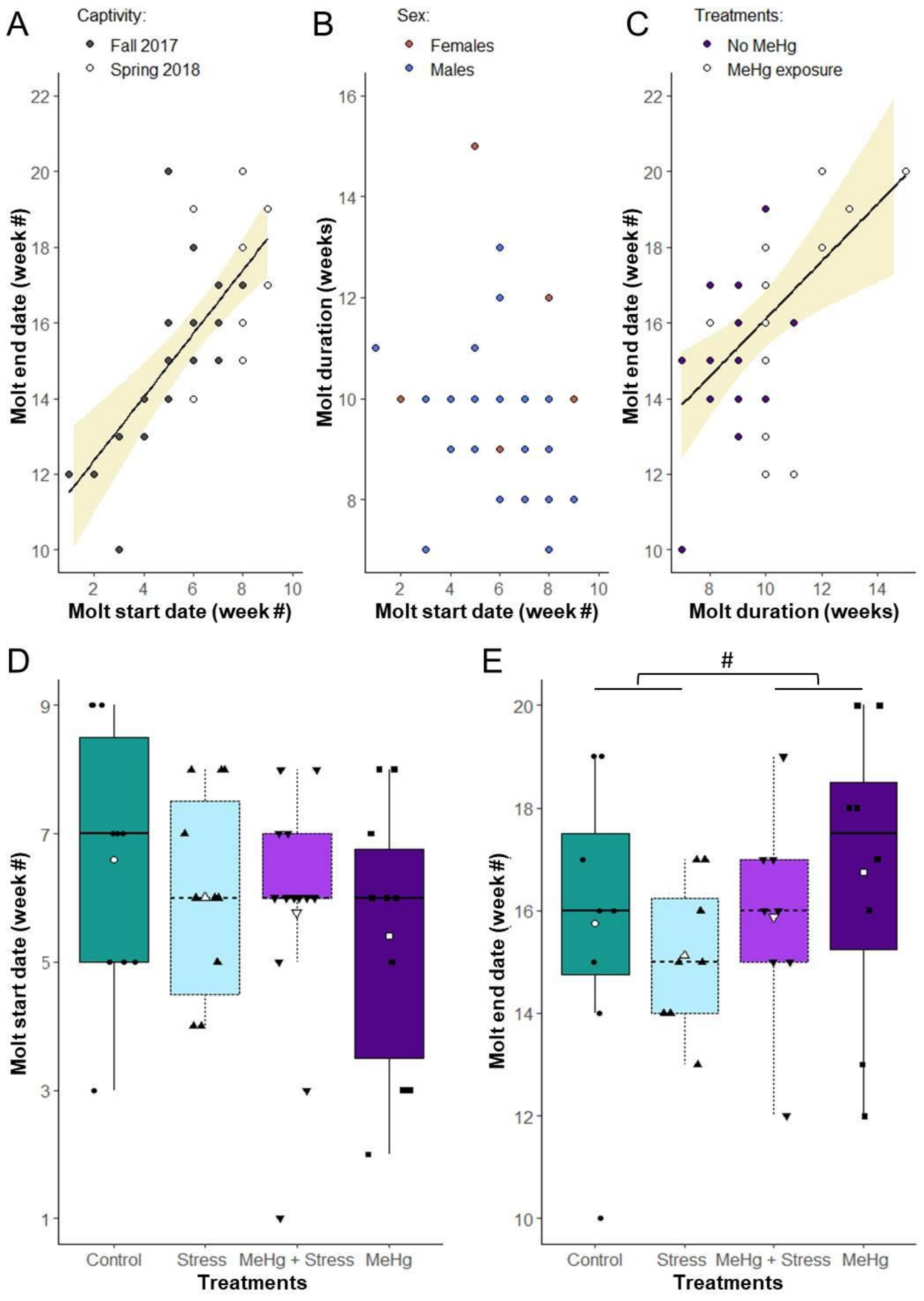

Fig. S3. Results from molt analysis, correlation and treatments effects.

A, B, C) Spearman correlations between molt measurements where the regression line and its standard error indicate the significant linear relation between measures ($p < 0.05$). (A) Correlation between molt start date and molt end date. To illustrated influential effect of bird's capture session, grey dots

Journal of Experimental Biology • Supplementary information

indicates birds caught in fall 2017 and white dots for birds captured in spring 2018. (B) Non-significant correlation between molt start date and molt duration (in weeks). To illustrated influential effect of bird's sex, blues dots represent male and red dots female birds. (C) Correlation between duration (in weeks) and molt end date. Dot colors illustrated influential effect of MeHg exposure with white circles representing unexposed groups (control and stress only treatment) and purple dots indicating exposed individuals (from MeHg only or co-exposure treatment).

D, E) Effect of food stress and MeHg exposure treatments on (D) molt start date, and (E) molt end date. Boxplots indicate 25th, 50th, and 75th percentiles and whiskers indicate range, with individual jittered data points overlaid. Boxplots color and symbols indicate treatment groups: control (dark blue with circles, $n = 8$), food stress (light blue with triangles, $n = 8$), combined MeHg and food stress (light purple with inverted triangles, $n = 8$), MeHg (dark purple with squares, $n = 8$). The white symbols indicate the mean molt duration for each treatment group. The # symbol indicates a non-significant MeHg treatment effect ($p < 0.1$).

Table S1. Selection of *lme* models predicting song sparrow's change in body condition[1] across time (ratio mass/tarsus used due to linear relationship between the variables[2]). The saturated model on body condition changes over time, included the triple interaction of stress, mercury and the quintic transformation of time as fixed effects, as well as the variable time and bird ID as uncorrelated random intercept and slope to account for the repeated measures. Note that the results from the last three time-points of body condition were published in a prior paper (Bottini et al., 2022), but here we analyzed the change in body condition throughout the whole experiment.

Part A shows the top-ranked candidate linear models (ΔAICc lower than two) predicting fat mass of song sparrows exposed to treatments groups (control, MeHg-only, unpredictable food stress only or combined MeHg and food stress) throughout the duration of the experiment (6 months). Reported in the table are the model degree of freedom (df), the log-likelihood (*logLik*), second-order Akaike's information criterion (AICc), difference in AICc between candidate models (ΔAICc), and proportional weight of each model (wi).

Part B shows model-averaged results of song sparrow body condition change. Square brackets indicate which level within a factor (e.g., stress [Y] = food stressed birds) is compared to the reference group (e.g., unstressed birds). The relative importance (RI) indicates the cumulative weight for each predictor given to models containing that predictor. A higher estimate indicates a stronger change in fat mass. Bold denote traits for which the 95% confidence interval (CI) surrounding the estimate does not overlap with zero.

A. Ranked candidate models

	df	*logLik*	*AICc*	*ΔAICc*	*wi*
stress + $Time^5$ + $Time^5$:stress	15	1105.40	-2179.86	0	0.69
MeHg + stress + $Time^5$ + $Time^5$:stress	16	1105.67	-2178.27	1.60	0.31
null	4	1054.75	-2101.42	78.44	$3.96.e^{-18}$

B. Parameter estimates of averaged top models

	RI	*Estimate*	*SE*	*95%CI*	*p*
**(Intercept)**	**-**	0.026	**0.0069**	**0.012 to 0.039**	**<0.001**
MeHg [Y]	0.31	0.0050	0.0068	-0.0084 to 0.018	0.47
Stress [Y]	1	0.0093	0.0091	-0.0086 to 0.027	0.31
**Time [1st degree]**	**1**	0.54	**0.084**	**0.37 to 0.70**	**<0.001**
**Time [2nd degree]**	**-**	0.13	**0.037**	**0.061 to 0.21**	**<0.001**
Time [3rd degree]	-	-0.020	0.036	-0.091 to 0.051	0.58
Time [4th degree]	-	0.028	0.036	-0.043 to 0.099	0.44
**Time [5th degree]**	**-**	0.16	**0.036**	**0.094 to 0.24**	**<0.001**
Time [1st degree]:Stress [Y]	1	0.014	0.12	-0.22 to 0.24	0.91
**Time [2nd degree]:Stress [Y]**	**-**	-0.16	**0.052**	**-0.26 to -0.057**	**0.0023**
**Time [3rd degree]:Stress [Y]**	**-**	-0.10	**0.051**	**-0.20 to -0.0043**	**0.041**
Time [4th degree]:Stress [Y]	-	-0.074	0.050	-0.17 to 0.025	0.14
Time [5th degree]:Stress [Y]	-	-0.0033	0.050	-0.10 to 0.095	0.95

[1] Despite being randomly assigned, at the start of the experiment, the stress-only treatment group had longer tarsus length than other treatments and higher body condition score than the combined exposure to stress and MeHg group. Also, females had lower body condition than males throughout the experiment. We hence standardized the differences by calculating the change in body condition compared to the pre-exposure time point set at a zero value. Overall, the differences between raw body condition and body condition change were qualitatively equivalent (see available R code).

[2] Because the ratio mass/tarsus assumes a linear relationship between variable, we first validated our use of this ratio as estimate of body condition. We created a linear model (lm) between the birds' mass

Journal of Experimental Biology • Supplementary information

and tarsus length including all the mass data collected across the experiment. The relationship between mass and tarsus of the birds was linear (lm tarsus: $F_{1,523}$ = 77.85, p = < 0.0001; lm model: residuals SE = 1.47; adjusted R^2 = 0.13). Additionally, change in raw body mass through time (compared to the body mass at pre-exposure time point set at a zero value) was also positively correlated with tarsus length (model including all the mass change data collected across the experiment; lm tarsus: $F_{1,523}$ = 19.037, p = < 0.0001; lm model: residuals SE = 1.10; adjusted R^2 = 0.03). This result pushed us to analyze change in body condition instead.

Table S2. Selection of *lm* models predicting song sparrow's molt start (A) and molt end (B). The saturated models' fixed effects included the interaction between stress and mercury as well as capture session (i.e., captured in fall 2017 or in spring 2018). Molt end model also included tarsus size as fixed effect. We followed the protocol described in the main manuscript to perform the candidate models selection and results extraction. See Table S1 for explanation of table notations.

**A. Molt start *lm* model**					
Ranked candidate models					
	df	*logLik*	*AICc*	*ΔAICc*	*wi*
Capture session	3	-79.75	166.11	0	0.57
Capture session + MeHg	4	-78.82	166.67	0.56	0.43
null	2	-90.39	185.072	18.97	$3.10.e^{-05}$
Parameter estimates of averaged top models					
	RI	*Estimate*	*SE*	*95%CI*	*p*
**(Intercept)**		**5.27**	**0.36**	**4.56 to 5.99**	**<0.001**
MeHg [Y]	0.43	-0.61	0.45	-1.52 to 0.31	0.20
**Capture session [2018]** [1]	**1**	**2.50**	**0.49**	**1.51 to 3.49**	**<0.001**
**B. Molt end *lm* model**					
Ranked candidate models					
	df	*logLik*	*AICc*	*ΔAICc*	*wi*
Capture session + MeHg + Tarsus	5	-63.78	139.86	0	0.67
Capture session + Tarsus	4	-65.88	141.23	1.37	0.34
null	2	-72.64	149.69	9.82	0.0029
Parameter estimates of averaged top models					
	RI	*Estimate*	*SE*	*95% CI*	*p*
(Intercept)		43.40	11.14	20.67 to 66.14	**<0.001**
MeHg [Y]	0.66	1.41	0.71	-0.049 to 2.87	0.058
**Capture session [2018]** [2]	**1**	**2.144**	**0.70**	**0.71 to 3.57**	**0.0033**
**Tarsus size** [3]	**1**	**-1.33**	**0.52**	**-2.39 to -0.27**	**0.014**

[1] Birds captured in spring 2018 started their molt later (mean ± SD molt start on week: 7.64 ± 1.08, n = 14) than birds captured in fall 2017 (5.13 ± 1.68; n = 30; Fig.S3A, S3D).

[2] Birds captured in spring 2018 finished their molt later (molt end on week 17.23 ± 1.74; n = 13) than birds captured in fall 2017 (14.95 ± 2.34; n = 19; Fig.S3A).

[3] Because molt end was influenced by tarsus size (mm) we tested if the size of birds was equally spread between treatments. We used a linear model on tarsus size as the independent variable and the interaction of stress with MeHg treatment as the dependent variable and we extracted the model's variables F-values via the *Anova* (type III) function. Bird's tarsus size was not different between MeHg exposed birds (*anova* of lm: $F_{1,40} = 0.075$, p = 0.79), food stress treatment (*anova* of lm: $F_{1,40} = 0.016$, p = 0.90) or interaction between treatments (*anova* of lm stress*MeHg: $F_{1,40} = 0.26$, p = 0.61) was explaining tarsus size. The model was equivalent to a null model (anova comparison with null model: $F_{3,40} = 0.26$, p = 0.63).

Journal of Experimental Biology • Supplementary information

Table S3. Selection of *lme* models predicting song sparrow's feathers mass (A) and feathers length (B). All saturated models included the triple interaction of MeHg, food stress exposure and primary feather number as well as sex as fixed effects and the birds ID as random intercept. We followed the protocol described in the main manuscript to perform the candidate models selection and results extraction. Indication of [Y] or [#] signal which factor group of the data (e.g., group exposed to mercury, primary feather #) is compared to the reference group (e.g., unexposed birds, primary P1). See Table S1 for explanation of table notations.

**A. Feather mass *lme* model**					
Ranked candidate models					
	df	*logLik*	*AICc*	*ΔAICc*	*wi*
Feather + MeHg+ Sex + Feather:MeHg	15	-41.37	115.75	0	0.51
Null	3	-207.27	420.68	304.92	$3.094e^{-67}$
Parameter estimates of best lme model					
		Estimate	*SE*	*95%CI*	*p*
(Intercept)		6.27	0.23	51.44 to 54.18	**<0.001**
MeHg [Y]		0.14	0.18	-0.53 to 1.60	0.43
**Sex [Males]**		**0.78**	**0.22**	**2.23 to 4.84**	**0.0014**
Feather [2]		0.062	0.082	0.91 to 1.97	0.45
**Feather [5]**		**1.42**	**0.084**	**4.41 to 5.30**	**<0.001**
**Feather [7]**		**1.41**	**0.082**	**2.22 to 3.089**	**<0.001**
**Feather [8]**		**1.20**	**0.084**	**-0.56 to 0.36**	**<0.001**
**Feather [9]**		**0.46**	**0.090**	**-7.57 to -6.50**	**<0.001**
**Feather [2]:MeHg [Y]**		**0.31**	**0.12**	**-0.51 to 1.011**	**0.0098**
Feather [5]:MeHg [Y]		0.12	0.12	-0.42 to 0.57	0.33
Feather [7]:MeHg [Y]		0.070	0.12	-0.51 to 0.42	0.55
Feather [8]:MeHg [Y]		0.014	0.12	-0.42 to 0.62	0.91
Feather [9]:MeHg [Y]		-0.20	0.13	-1.049 to 0.54	0.14
**B. Feather length *lme* model**					
Ranked candidate models					
	df	*logLik*	*AICc*	*ΔAICc*	*wi*
Feather + MeHg+ Sex + Feather:MeHg	15	-246.72	526.35	0	0.27
Feather + MeHg+ Sex	10	-252.69	526.67	0.32	0.23
Feather + MeHg	9	-253.99	527.026	0.68	0.20
Null	3	-503.96	1014.062	487.71	$3.41e^{-107}$
Parameter estimates of averaged top models					
	RI	*Estimate*	*SE*	*95% CI*	*p*
(Intercept)		52.80	0.70	51.43 to 54.17	**<0.001**
MeHg [Y]	0.72	0.74	0.51	-0.26 to 1.73	0.15
**Sex [Males]**	**1**	**3.54**	**0.66**	**2.23 to 4.84**	**<0.001**
**Feather [2]**	**1**	**1.44**	**0.27**	**0.91 to 1.97**	**<0.001**
**Feather [5]**		**4.85**	**0.23**	**4.41 to 5.30**	**<0.001**
**Feather [7]**		**2.65**	**0.22**	**2.22 to 3.089**	**<0.001**
Feather [8]		-0.10	0.23	-0.56 to 0.36	0.66
**Feather [9]**		**-7.034**	**0.27**	**-7.57 to -6.50**	**<0.001**
Feather [2]:MeHg [Y]	0.39	0.64	0.37	-0.079 to 1.36	0.081
Feather [5]:MeHg [Y]		0.16	0.37	-0.55 to 0.92	0.62
Feather [7]:MeHg [Y]		-0.13	0.37	-0.85 to 0.60	0.73
Feather [8]:MeHg [Y]		0.25	0.38	-0.49 to 1.00	0.51
Feather [9]:MeHg [Y]		-0.66	0.39	-1.43 to 0.11	0.094
